# Empirical Analysis of the Dynamics of the COVID-19 Epidemic in Urban Embedded Social Networks

**DOI:** 10.3389/fpubh.2022.879340

**Published:** 2022-06-02

**Authors:** Zihao Wang, Yue Zhuang, Chao Fan

**Affiliations:** School of Safety Science and Emergency Management, Wuhan University of Technology, Wuhan, China

**Keywords:** COVID-19, social network, contact tracing data, urban epidemic, Nanjing

## Abstract

**Background:**

Due to the continual recurrence of COVID-19 in urban areas, it is important to know more about the evolution of the epidemic within this setting to mitigate the risk of the situation getting worse. As the virus spreads through human society, the social networks of confirmed cases can provide us with crucial new insights on this question.

**Methods:**

Based on the epidemiological reports of 235 COVID-19 cases in Nanjing, we constructed a social contact network for the epidemic. By analyzing the structure of this network, we explored the transmission characteristics of the epidemic, to provide evidence-based explanations for its transmission.

**Results:**

In our constructed transmission network, more than half (95/165, 57.58%) of patients were found not to have transmitted the infection, with only 15 (9.10%) source patients accounting for more than a third of the contagion (60, 36.36%), suggesting that the transmission of COVID-19 varies per individuals. Patients in the 31 to 50 age group were the main source of infectious clusters, with females playing a more active role in passing on the infection. Network component analysis identified nine components with disproportionate concentrations of influential patients, accounting for 49.09% (81) of the patients and 59.09% (78) of epidemiological network contacts. Family aggregation may favor disease transmission, and parenthood is the relationship with the highest infection risk within the family cluster. In addition, some specific public places, such as chess and card parlors, were found to be notable hotspots for community infection.

**Conclusion:**

This study presents the evolution of the urban epidemic from the perspective of individual-level and socially interactive processes. This real-world evidence can help to increase public awareness of the epidemic, formulate countermeasures, and allocate limited public health resources for urban management.

## Introduction

Even though Chinese authorities never let their guard down since the beginning of the coronavirus disease 2019 (COVID-19) crisis and the current prevention and control measures are implemented soundly in China, the resurgence of the epidemic has been noted in various cities across the country. Therefore, it is important to understand the evolution of the epidemic in a city if we are to mitigate its risk. On this issue, the social and urban fundamental principles of the transmission of COVID-19 deserve our attention (1). In the case of such infectious diseases, the virus is transmitted among people through their social contacts (2). From this perspective, the evolution of an urban epidemic can be seen to be the same as the formation of social contact networks among those with the virus. In this way, analyzing the social contact networks of confirmed cases helps to identify the transmission characteristics of COVID-19 in cities; this is of vital importance in formulating countermeasures and in allocating limited public health resources for urban management (3).

Since the outbreak of COVID-19, many mathematical models of disease transmission have been used to predict the evolution of urban epidemics and to design interventions; these have included regression models, Susceptible-Infected-Recovered (SIR) models, and their evolutionary branch models (4–6). Furthermore, other variables were added to expand the explanatory scope of these models. For instance, to counter the wide dissemination of false information related to the epidemic, researchers appends individual communicating willingness and forgetting effects to the Susceptible-Exposed-Infected-Recovered (SEIR) model to study the mechanisms by which public opinion is formed, disseminated, and polarized (7). In addition, many advanced statistical techniques, such as Bayesian analysis, have been used in the study of epidemics (8). The use of multiple models enriches the research in this field. However, the above studies mostly focus on either predicting or retrofitting the epidemic situation, while failing to show the dynamics of disease transmission from the perspective of individual level (9). For this reason, prediction results using mathematical modeling can sometimes be very different from the actual numbers that are subsequently reported (10, 11). Therefore, exploration of the microscopic spread mechanisms of viruses is urgently needed.

Understanding the changes in the network itself and the role of human adaptive behavior in the process of disease transmission can provide effective insight into the spread of viruses in a real-life network (12). Previous studies of the epidemic pattern of infectious diseases, considering the contact network have demonstrated the potential of social network analysis in explaining the real-world phenomena. For example, using dynamic bipartite graphs to simulate smallpox outbreaks in urban social networks, Eubank et al. found that these outbreaks can be contained by a strategy of targeted vaccination combined with early detection without resorting to mass vaccination of the population (13). Block et al. adopted a social network approach to evaluate the effectiveness of distancing strategies designed by network topology to demonstrate that a strategic reduction of contact through social networks strongly enhances the effectiveness of social distancing measures while keeping risks low (14). A consideration of the heterogeneity of contact patterns in epidemics by Li et al. used a contact network model to more realistically simulate two stages of the COVID-19 outbreak on the “Diamond Princess” cruise ship (15). All the studies above used different contact network models to explain the outbreak more accurately, however, the network models used in these studies are generally based on theory or simulation and lack disease transmission data from the real world.

To understand the evolution, prevention, and control of the epidemic, it is vital to analyze the spatial and temporal co-occurrence of confirmed cases (9). The support of real-world disease transmission data ensures the accuracy and efficacy of non-pharmacological interventions (12). In addition to macro-level prevention and control measures, it is also important to provide guidance and regulation on individual behavior, which requires information on the spread of infection through social contact (9). Encouragingly, the spatiotemporal information contained in daily case reports, including the relationship between different cases and their movement trajectories, provides an important source of real-world data on the evolution of urban epidemics at the individual level (16). For COVID-19, some researchers have used real-world case data to conduct studies of contact networks. By analyzing empirical, interpersonal, and physical contact networks using mobile device data in the city of Portland, Oregon, the impact of these different network topologies on the spread of COVID-19 was identified (17). Using contact tracing information of 135 and 143 confirmed patients with COVID-19 in Tianjin and Chengdu, respectively, from January 21 to February 22, 2020, researchers were able to trace the transmission source and contacts, assess the current situation of transmission and prevention and provide evidence for the response and control of the COVID-19 epidemic in other regions in the world (3). These studies deepen our understanding of epidemic transmission and help us to formulate targeted measures based on scientific analysis of transmission networks.

This study collected all confirmed COVID-19 cases published in Nanjing, China, from July 20 to August 26, 2021, a total of 235 cases. By constructing the social contact networks of these cases based on their records, this paper explores the structural characteristics of the COVID-19 transmission network. We attempt to explain these characteristics using factual evidence to provide a scientific basis for increasing public awareness and formulating prevention and control measures.

## Methods

### Data Source

On July 20, 2021, a new local wave of COVID-19 was identified in Nanjing, which spread to more than 20 cities in Beijing, Hunan, Anhui, Liaoning, and other provinces. Previously, there had been no local cases of COVID-19 in the city for more than a year. Investigations revealed that the source of the virus was an inbound flight from Russia; a cleaner was infected, which subsequently resulted in community transmission in the city. The specific and unique primary patient clarifies the evolution of the epidemic in this case. Since August 26, Nanjing has reported 235 cases of COVID-19. However, there were no new confirmed cases for the 2 weeks from August 13 to 26. For this reason, we believe that this wave of the epidemic was a complete transmission event involving the whole process of emergence, development, and extinction. This event is a perfect candidate from which to gain relevant empirical evidence on urban epidemic evolution.

Nanjing is the epicenter of this outbreak, and the epidemiological investigation reports were quickly communicated by the Nanjing Health Commission. Nanjing was, therefore, selected as the representative study area for this research. All the samples in this study are collected from the official websites of the Nanjing Health Commission from July 20 to August 26, 2021. Structured information about the samples is available in the Supplementary Materials section.

### Study Design

First, we extracted relevant demographic and spatiotemporal information from individual records in the reports published by the city's official health committee to build a structured dataset that included sex, age, household location, occupation, activity tracking, the relationship between cases, and time of definite diagnosis.

Second, we constructed the transmission network using the following two steps. First, each patient was represented as a node, with their medical record number provided by the daily bulletins used as a unique identification number. Then, the epidemiological contacts among cases were identified and represented as directional edges emanating from a “source patient” to a “target patient” to link those nodes. Based on the information about household location, place of employment, activity track, and social relationship, we determined the spatial and temporal co-occurrence of confirmed cases, which suggested the existence of the epidemiological contacts among them. In this way, we hypothesized that the first confirmed cases are the source of infection for their spatial and temporal co-occurrence persons. Cases without a clear exposure history were scattered outside the transmission network and were not part of it.

Third, we visualized and analyzed the constructed network. We imported the adjacency matrix of the network into Gephi software (Version 0.9.2); the Force atlas layout and Fruchterman Reingold layout in Gephi software were applied to visualize the transmission network. We analyzed the network attributes generated by Gephi using MS Excel to explain the transmission characteristics of the infection network in the context of known facts. Then, the social relationships among cases were embedded in the network to provide further insight on infection spread.

### Measures

We performed a conventional demographic analysis of the study data that were also the basis of our network research. Then, we analyzed the transmission network in three ways: whole network, nodes and components, and social relations. Some studies have shown that network attributes are closely related to the spread of COVID-19 in the network (18, 19). Insight into social relationships among confirmed cases could help identify the high-risk contact. We calculated the relevant network indicators such as network diameter, average path, and centrality measures to assess the key characteristics that were influential in transmitting the infection. Table 1 lists the details of the indicators. The network used for analysis is displayed in Supplementary Figure 1.

**Table 1:** Social network measures.

**Level**	**Indicator**	**Definition**	**Equation**	**Implication in COVID-19**
Whole network	Number of nodes	The number of nodes in the network	*N* = |*n*|	The number of patients in the transmission network
	Number of edges	The number of edges in the network	*E* = |*e*|	The number of epidemiological contacts among patients
	Network density	The number of existing ties between nodes, divided by the number of possible ties	$D_{e}=\frac{E}{N\left(N-1\right)}$	The number of existing epidemiological contacts among patients, divided by the number of possible epidemiological contacts
	Mean path length	The average of the shortest path length between all possible node pairs	$L=\frac{2}{N\left(N-1\right)}\underset{i\neq j}{\sum }d_{ij}$	The average epidemiological contact distance between the source and target patients
Nodes and components	Outdegree centrality	The number of links to target nodes from a source node	$C_{out}\left(n_{i}\right)=\overset{N}{\underset{j=1}{\sum }}a_{ij}$	The number of secondary patients of a source patient
	Indegree centrality	The number of incoming links to a node from source nodes	$C_{in}\left(n_{j}\right)=\overset{N}{\underset{i=1}{\sum }}a_{ij}$	The number of epidemiological contacts incident upon a patient from source patients
	Betweenness centrality	The ability of a node to lie on a geodesic path between other pairs of nodes in the network	$C_{b}\left(n_{i}\right)=\underset{j}{\sum }\underset{k}{\sum }g_{jk}\left(i\right)$	The ability of a patient to act as a bridge in the transmission of the virus
	Network component	The islands of interlinked nodes that are disconnected from other nodes of the network	–	Connected structures with interlinked patients, but disconnected from other similar components in the network
Social relations	Family	–	–	Family relationships between patients including parentage, couple and other kin
	Community life	–	–	The epidemiological contacts occurring in community life
	Colleague and friend	–	–	Colleague or friend relationships between patients

## Results

### Demography

**Figure 1:**
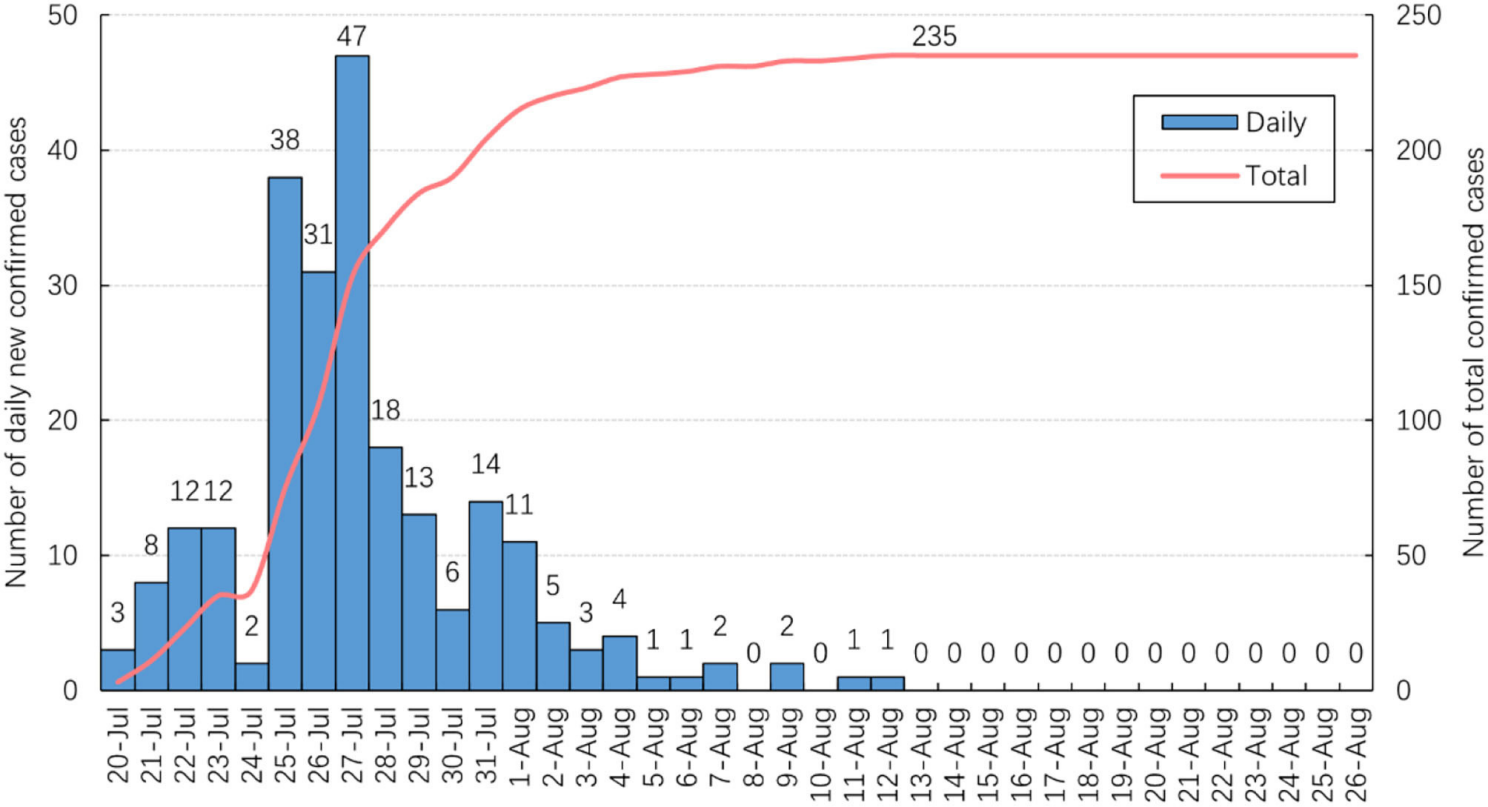
The change in the number of confirmed cases over time.

As shown in Figure 1, 47 new confirmed cases were reported on July 27, the highest daily total since July 20. The number of new daily cases decreased and did not exceed two after August 5. No new cases were reported from August 13 and the total number of confirmed cases stabilized at 235.

**Figure 2:**
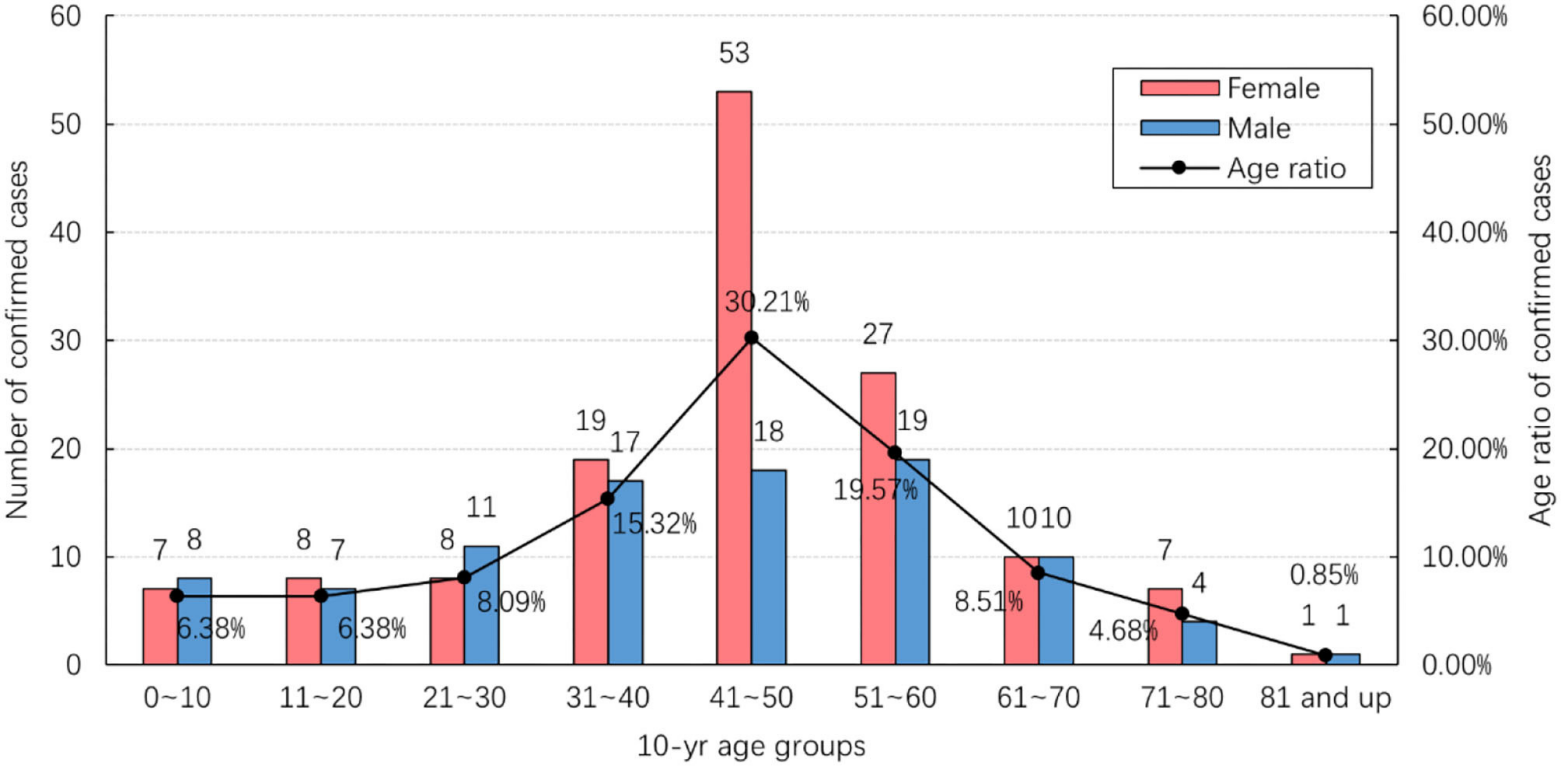
Age and gender distribution of confirmed cases.

Figure 2 shows the age and gender distribution of the study data. Of the total 235 confirmed cases, 95 (40.43%) were male and 140 (59.57%) were female. They ranged in age from 4 months to 87 years, with an average age of 43.12 years. Most cases (71/235; 30.21%) were found in the 41–50 age range. The number of female cases in this age group was significantly larger than that of male cases, while there was no significant difference in other age groups.

### Whole Network

In our constructed transmission network, there were 165 (70.21%) nodes related to 165 confirmed cases with epidemiological contact with others. The remaining 70 (29.79%) nodes were isolated patients without clear epidemiological contact information. The number of network edges was 132, that was, 132 pairs of contact relationships existed between 165 patients. Our network density was measured as 0.005 and the mean path length between the patients was 1.600 (Table 2).

**Table 2:** The calculation result of whole network measures.

**Measure**	**Result**
Number of nodes	165
Number of edges	132
Network density	0.005
Mean path length	1.600

### Nodes and Components

Table 3 shows the summary statistics of node attributes. There were 95 (57.58%) nodes with zero outdegrees, which means that these 95 patients did not infect other people. Of those remaining, 70 (42.42%) nodes had an outdegree range of 1~7 and were the source of infection to 132 (80%) targets. Among these source patients, 15 individuals had infected more than two people, and these collectively led to 36.36% of all secondary infections in the network.

**Table 3:** Summary statistics of node attributes.

**Node attributes**	**Range**	**Mean (S.D.)**	**Value (No of nodes, Per cent %)**	**95th percentile**
Outdegree	0~7	0.800 (1.266)	0 (95, 57.58), 1 (38, 23.03), 2 (17, 10.30), 3 (8, 4.85), 4 (2, 1.21), 5 (3, 1.82), 6 (1, 0.61), 7 (1, 0.61)	5
Indegree	0~5	0.800 (0.564)	0 (40, 24.24), 1 (121, 73.33), 2 (3, 1.82), 5 (1, 0.61)	6
Betweenness	0~26	0.764 (3.096)	0 (140, 84.85), 1 (8, 4.85), 2 (4, 2.42), 3 (5, 3.03), 4 (2, 1.21), 6 (2, 1.21), 12 (1, 0.61), 18 (1, 0.61), 19 (1, 0.61), 26 (1, 0.61)	1

There were 40 (24.24%) patients with an indegree centrality measure of zero, therefore, having no epidemiological contact with a source patient. The number of patients who had an indegree of one respectively corresponding to a source patient was 121 (73.33%). The remaining four (2.42%) patients had an indegree of more than one, implying multiple sources were identified for each of them. Most contagions occurring in this epidemic round were one on one.

The range of betweenness was 1~26 for 25 (15.15%) patients, and they played a bridging role in the transmission of infection. Disproportionately, 140 (84.85%) patients with zero betweenness centrality did not bridge the transmission.

As shown in Table 4, women had a higher mean outdegree and a higher betweenness than men (0.990 vs. 0.529, 1.113 vs. 0.265 respectively). Patients ranging in age from 31 to 50 also had a higher outdegree, indicating that this age group was the main factor causing the seeding of clusters. Most of the age group had a high value of betweenness centrality. However, it was relatively low in the 41 to 50 age group, which means that people in this age group seldom serve as a bridge of infectious transmission. It is interesting to note that all patients older than 70 years had zero outdegrees and betweenness; therefore, they did not infect other people in this outbreak.

**Table 4:** Mean outdegree and betweenness by sex and age group.

**Age groups**	**Mean outdegree**			**Mean betweenness**		
	**Women**	**Men**	**Combined**	**Women**	**Men**	**Combined**
0~10	0.143	0.375	0.267	0.857	0.750	0.80
11~20	0.667	0.000	0.333	0.667	0.000	0.333
21~30	0.857	0.600	0.706	1.714	0.100	0.765
31~40	1.571	1.000	1.381	1.786	0.571	1.381
41~50	1.481	1.091	1.368	0.222	0.000	0.158
51~60	0.947	0.615	0.813	1.684	0.538	1.219
61~70	0.500	0.000	0.278	2.300	0.000	1.278
71 or over	0.000	0.000	0.000	0.000	0.000	0.000
Combined	**0.990**	**0.529**	**0.800**	**1.113**	**0.265**	**0.764**

The aggregate network consisted of 39 components, most of which occurred between July 25 to 28 (Supplementary Figure 2). The peak production of the components took place on July 26. Components were not occurring consecutively, rather, they were mostly simultaneous over time. Of these 39 components, nine components, each consisting of six or more nodes, accounted for 49.09% (81) of patients and 59.09% (78) of epidemiological contacts in the network. All patients who had higher outdegree and betweenness centrality measures (ranking 97.5 or more) featured disproportionately in the nine components. The identified network component structure highlights that not all infections were directly transmitted from the primary patients to many target patients, but predominantly through influential patients with high outdegree or betweenness who transmitted the infection from them (Table 5).

**Table 5:** Major network components identified in the transmission network.

**Network components^**a**^**	**No & % of patients in components**	**No & % of transmission contacts in components**	**Component initiation date^**b**^**	**Patients with out degree centrality ≥97.5th percentile**	**Patients with betweenness centrality ≥97.5th percentile**
C1 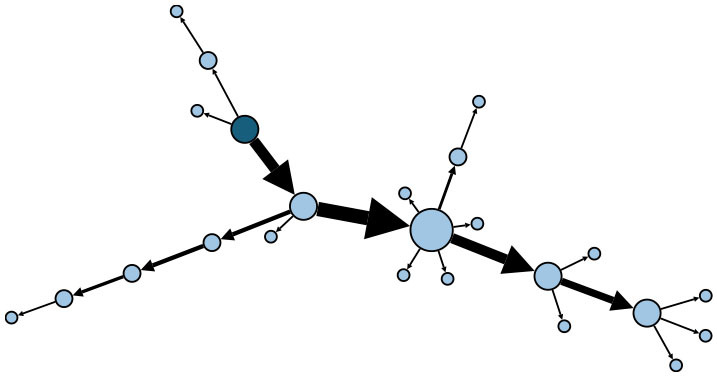	24 (14.55)	23 (17.42)	July 21	P30	P28, P30, P74, P83, P134, P145
C2 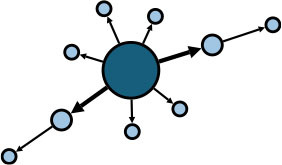	10 (6.06)	9 (6.82)	July 25	P44	–
C3 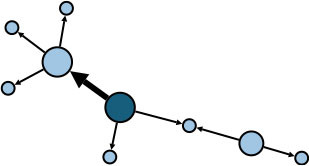	9 (5.45)	8 (6.06)	July 25	–	–
C4 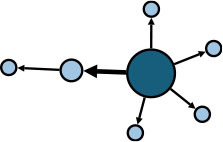	7 (4.42)	6 (4.55)	July 20	P4	–
C5 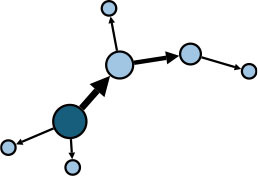	7 (4.42)	6 (4.55)	July 23	–	–
C6 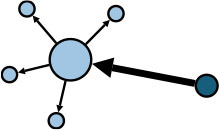	6 (3.64)	5 (3.79)	July 22	–	–
C7 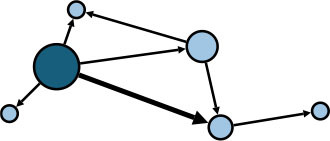	6 (3.64)	7 (5.30)	July 22	–	–
C8 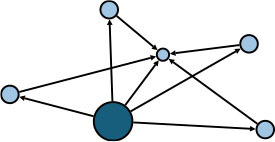	6 (3.64)	9 (6.82)	July 25	P39	–
C9 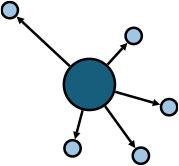	6 (3.64)	5 (3.79)	August 4	P225	–
C1-C9 summary	**81 (49.09)**	**78 (59.09)**	**July 20 to August 4**	**5 patients**	**6 patients**

a *In the sketch of network components, darker nodes represent the first diagnosed source patient of a components, light nodes represent target patients. Node size is determined by outdegree. Edge thickness is determined by beteenness of parent node*.

b *Component initiation date is the diagnosis date of the primary patient in that component*.

### Social Relations

**Figure 3:**
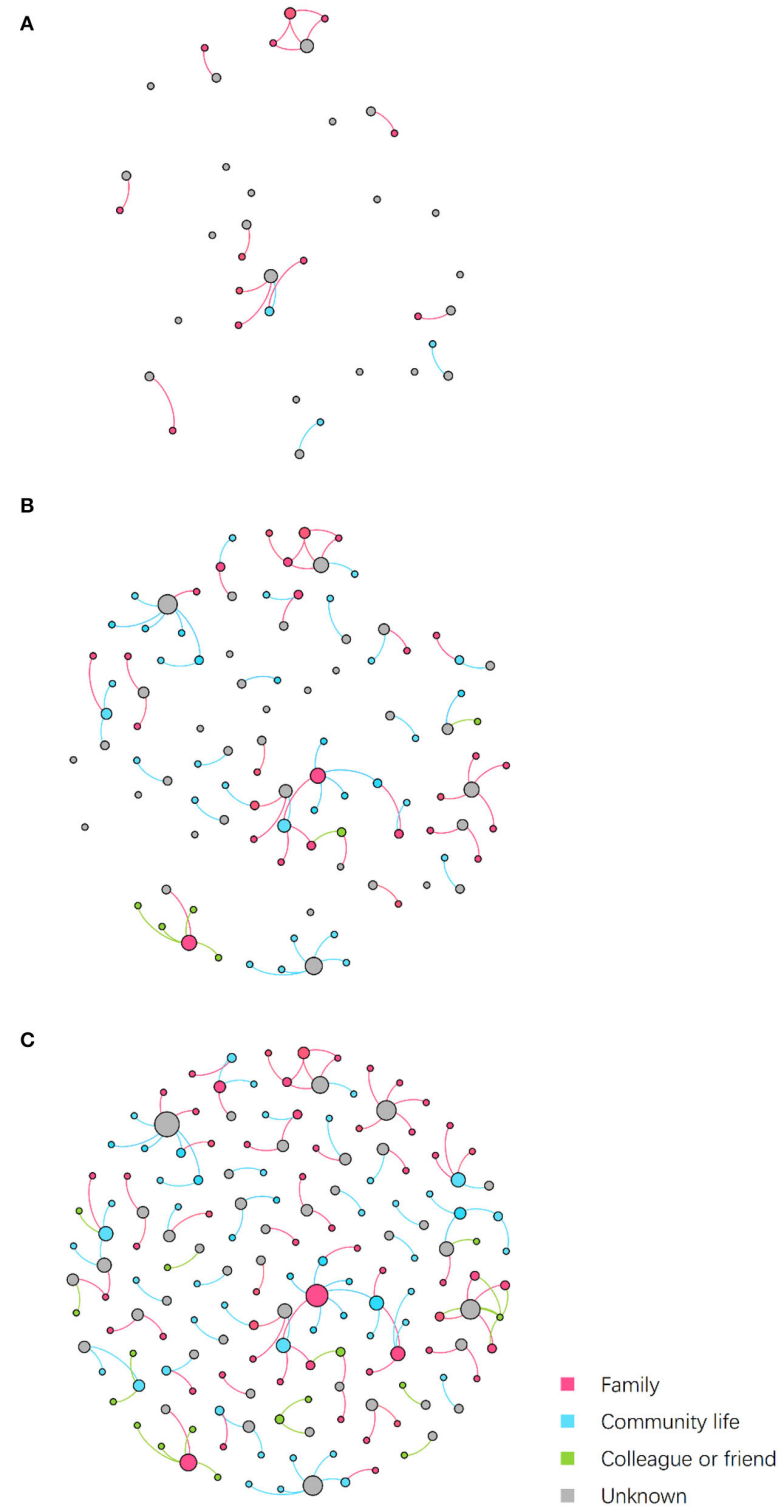
Dynamic contact network embedded social relations. Nodes represent patients, while edges represent transmission routes between patients. The color of edges represents the social relations between nodes connected by epidemiological contacts. The color of nodes represents the kind of social contacts by which they were infected. Node size is determined by outdegree. **(A)** Network on July 24. **(B)** Network on July 27. **(C)** Network on August 12.

Figure 3 shows the embedded social relations of the dynamic contact network. Three time points were identified to represent the network structure: the early period (July 24), the outbreak period (July 27), and the epilog period (August 12). The early period network was loose, with the spread of infection mainly due to family contact. During the outbreak period, community transmission increased, and several clusters appeared. In the epilog period, clusters continued to develop based on those formed in the previous stage, with few new large clusters developing, which indicates that the control measures taken were effective. Infection through contact between colleagues or friends was found to be more frequent than in the initial two stages of the epidemic.

**Figure 4:**
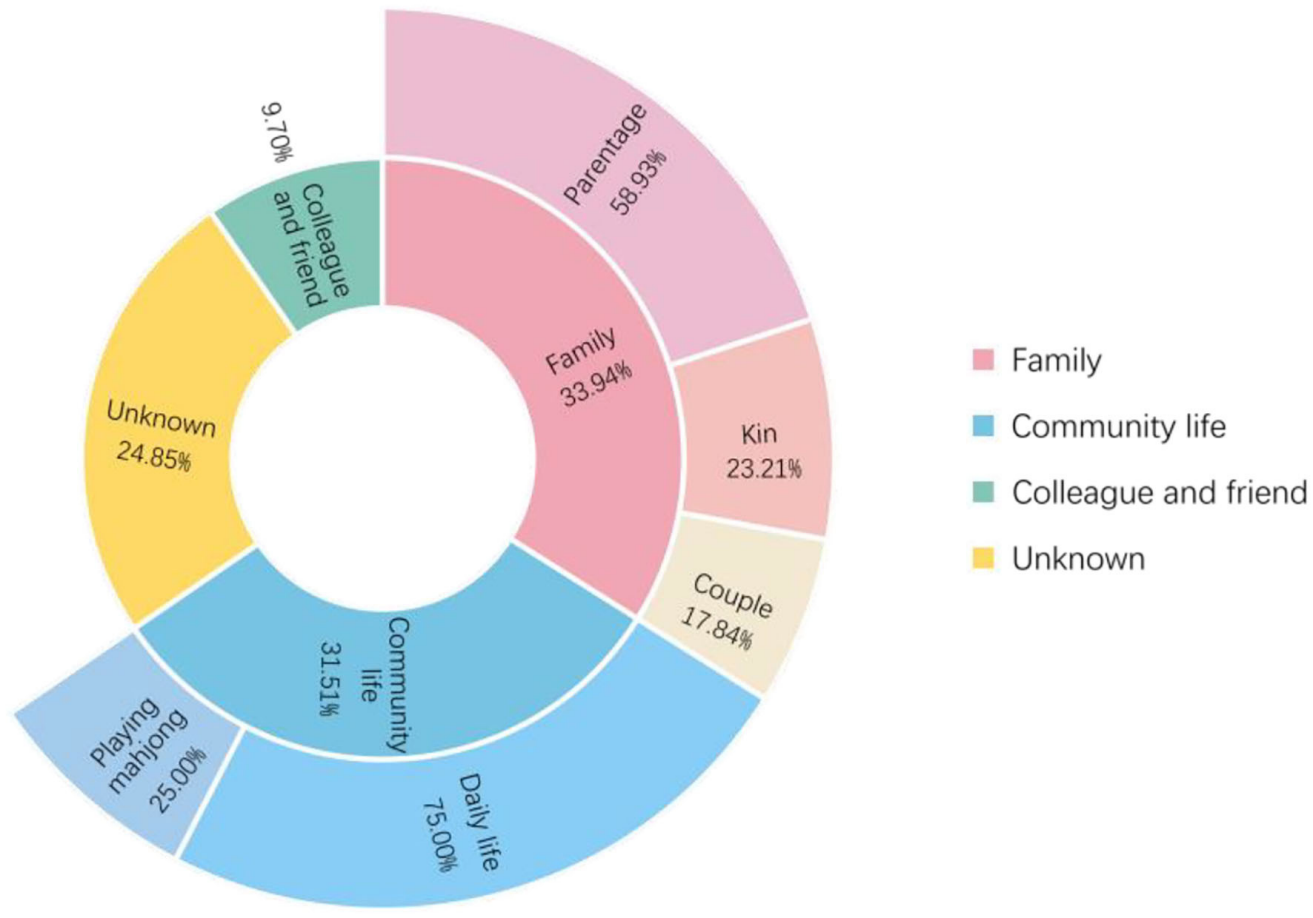
Statistics for social relations through which cases were infected.

As shown in Figure 4, of the 165 patients in the transmission network, 56 (33.94%) patients were infected through family clusters, involving 37 households. In Figure 3, these patients form a cluster of three or more nodes, showing the clustering characteristics of a “small world” (20). In a family cluster, parentage is the relationship with the highest infection risk, as 58.93% of contagions occurred between parents and children. By contrast, only 17.86% of contagion was transmitted between couples. A total of 52 (31.52%) patients were infected through community living. Most cases were caused by contact with Lukou airport employees in the community, including talking with neighbors, shopping at supermarkets, and playing mahjong. Furthermore, some specific public places deserve our attention, especially chess and card parlors, which became hotspots for community infection. A quarter of patients infected in the community were exposed through playing mahjong. Only 16 (9.70%) cases were infected by colleagues or friends. About 41 (24.85%) patients had unclear infection pathways because their records did not provide enough information to trace the full transmission chains.

## Discussion

Demographic data show that most COVID-19 cases in Nanjing were middle-aged and female. The number of female cases was significantly larger than that of males in the 41 to 50 age group, while there was no significant difference in other age groups. The hotspot population of this outbreak was the cleaning staff at Nanjing Lukou Airport who were mostly females aged between 41 to 50, which may account for the differences described above in age and gender distribution.

The network density was very low at only 0.005, indicating that the transmission network was loosely connected. In this case, each edge is closely related to the connectivity of the network, and cutting off some connections may break the network up into several isolated sub-networks. The low network density provides hard evidence of the reliability of social distancing. From this, avoiding unnecessary social contact during an epidemic will help to limit the spread of the virus to a small group of people. A calculation of mean path length indicated that on average any source and target patients in the network could be reached by crossing 1.6 steps. This suggests that the transmission of infection is limited to less than two steps on average. From a social network perspective, the dynamics of the infection transmission are closely related to the concept of path lengths, which indicates the number of network steps needed to connect two nodes. A short mean path length will facilitate the spread of the virus on the network. Consequently, one aim of social distancing should be increasing the average network distance between individuals by smartly and strategically manipulating the structure of interactions (besides the general reduction of contact) (14).

The fact that while more than half (95, 57.58%) of the patients did not transmit infection, only 15 (9.10%) source patients, accounted for more than a third of contagion (60, 36.36%), suggests an individual-level variation in the transmission of COVID-19. A study found that the estimate of the degree of heterogeneity can be biased downward by small sample size or under-reporting of zero-class events but are not biased upward by any of the factors considered (21). From this, the actual result might be more disproportionate than it is now since many patients were excluded from the network. Similar transmission patterns are reported in other areas in China, such as Shenzhen where 8.9% of the cases caused 80% of all infections and Hunan where 80% of secondary infections were traced back to 15% of primary infections (22, 23). The similarity in these results suggests that our findings may apply universally within the population. The individual-level variation in the transmission of COVID-19 indicated by our network analysis methods serves as a reminder to the government to focus intervention efforts on identifying key nodes, namely super-spreaders, and controlling them in a targeted manner.

**Figure 5:**
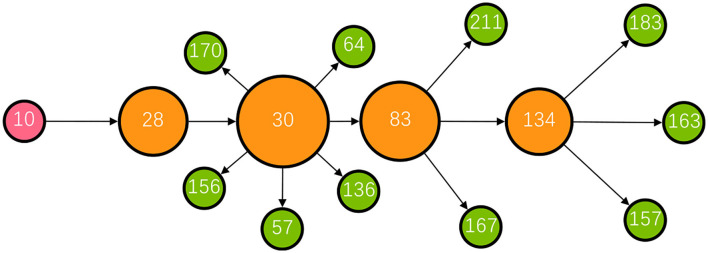
The transmission chain involving P30. The red node represents the primary patient in the transmission chain; the orange nodes represent bridging patients; the green nodes represent target patients. The larger the node size, the greater the betweenness centrality of the patient.

Except for the minority of influential source patients such as super-spreaders, bridging patients who transmitted the infection from these influential source patients were crucial in the development of a transmission network, for example, patient 30 (P30) with a high betweenness centrality measure of 26. Figure 5 shows the transmission chain involving P30; she was infected by her daughter-in-law (P28) who had been in contact at a gym with a cleaner at Nanjing Lukou Airport and subsequently transmitted infection to six other individuals at a chess and card parlor. P83, one of the six target patients, then transmitted the infection directly or indirectly to six other individuals. In this transmission chain, P30 acted as a bridge to diffuse the virus from the source to the city community and was responsible for 12 secondary infections. Even though susceptible people may never have had contact with primary patients, they could establish connections with them through these influential bridging patients and unwittingly become a similar transmission bridge to spread the virus. These actors may not spread the infection to many contacts, but their bridging characteristic accelerates transmission in the community (24). This is particularly the case for cross-community bridges, for example, taxi or ride-hailing drivers who travel around the city. To reduce the transmission efficiency of the virus around the network, we should not only contain the source but also focus on bridges on the critical transmission path.

In our study, patients in the 31 to 50 age group were the main force behind seeding the clusters, with females playing a more active role in bridging the infection, possibly reflecting the close community ties between them. Public health authorities could prioritize both these individuals and the clusters for rigorous containment and adopt control measures to the transmission characteristics of specific actors. Further study is required to determine what social and behavioral characteristics of these groups result in these differences.

Analysis of the network components highlights that their formation and development play a decisive role in the scale of the transmission network. As nine components made up the majority (59.09%) of the total transmission contacts, it is clear that a few major components constitute the main body of the network. In addition, influential patients (with higher outdegree or betweenness) are predominantly found in these major components, which may mean that influential patients connecting as small components may contribute to much of the transmission events when compared to sole influencers or superspreaders (25, 26). Therefore, to limit the size of the network, it is necessary to prevent cases from forming a giant component in the contact network and restrain the formation and development of several major components, that is, to avoid the occurrence of cluster infections in practice.

Family aggregation may favor disease transmission and parentage is the relationship with the highest infection risk within a family cluster. The family has specific importance in the context of infectious disease dynamics (27). Several epidemiological studies have reported that a substantial amount of COVID-19 transmission occurs within households (28, 29). Family is the basic clustering unit of any society, and it is difficult for family members to effectively isolate themselves from other members. Good hygiene awareness and habits for family members are essential during the normalization stage (30). The study shows in-house quarantine is not effective, therefore, building temporary housing units to quarantine suspected infections is necessary to limit the spread.

In addition, community infection cannot be ignored, particularly in public places such as chess and card parlors, which were infection hotspots in this outbreak. Mahjong, a popular pastime among older Chinese people, is usually played close to each other in crowded, poorly ventilated rooms. Furthermore, the elderly is a high-risk group, who are vulnerable to the virus (31). In one instance, P30, a 60-year-old female, frequently visited a chess and card parlor from July 16 to 20, during which time she transmitted the virus from her daughter-in-law to six other people. Therefore, such densely crowded places with frequent close contact need to be managed and controlled. As discussed earlier, as the majority of individuals do not contribute to transmission, there is benefit in preventing relatively rare superspreading events. Identifying factors and the characteristics of settings, such as chess and card parlors that could lead to these outbreaks, will play a key role in designing effective control strategies (32, 33). Routine temperature checks and Health Code verification should be required for anyone seeking to enter these places to prevent admission to suspected virus carriers, and the number of visitors should be restricted to limit personnel aggregation. In addition, it is worth noting that most patients had used taxis or ride-hailing. Moreover, two of the reported cases were taxi drivers. Due to the nature of this occupation, drivers usually have a wide urban mobility range, and they can easily spread the virus across a region. Hence, the management department should impose more strict epidemic prevention regulations for taxis and ride-hailing, requiring drivers to disinfect, ventilate their vehicles, and protect their health.

We also noticed that some patients (41, 24.85%) had an inconclusive infection pathway into the network. An unbroken transmission chain plays an important role in epidemic prevention and control (34). For example, by constructing transmission chains, researchers found that the spread of COVID-19 in the northern region of Italy had occurred earlier than February 20, 2020, when the first COVID-19 case was confirmed in the Lombardy region (35). This highlights the fact that contact tracing efforts should be further improved in China.

Due to data limitations, there are still some shortcomings in this study. The contact network only can capture part of the dynamic transmission process of COVID-19 as contact relationships were not fully reported in some cases. Therefore, future studies can conduct relevant analyses based on more complete and comprehensive data that can be acquired from other database sources. Of course, this requires a lot more effort in epidemiological investigations. From this, we suggest that the exposure history of each infected individual is recorded as completely as possible by health administrators and a uniform format is developed for daily reports to show structured data. In the process, attaching great importance to privacy protection is required. Policymakers should develop a standard for processing and releasing such data. Also, the privacy rights holders should be provided with the opportunity to participate in the negotiation. These efforts can assist in tracing integrated transmission networks and improving awareness of these in COVID-19 epidemics.

## Conclusion

By constructing and analyzing the contact networks of confirmed COVID-19 cases, we show the evolution of the epidemic in the city from the perspective of individual-level and social interaction processes. Our study indicates that virus transmission is closely related to urban life and social interaction, which suggests more attention should be focused on fundamental social and urban factors in the transmission of COVID-19, and not only the biological features of the virus itself. Evidence from real-world examples is used to explain this to help increase public awareness of the epidemic, formulate countermeasures, and allocate limited public health resources for urban management.

## Data Availability Statement

The original contributions presented in the study are publicly available. This data can be found here: [http://wjw.nanjing.gov.cn/njswshjhsywyh/?id=xxgk_228].

## Ethics Statement

The study used anonymized, secondary data released publicly on official websites, and does not require ethical approval or consent in accordance with local legislation and institutional requirements.

## Author Contributions

ZW: data curation, methodology, formal analysis, investigation, and writing-original draft. YZ: methodology, supervision, and writing-review and editing. CF: writing-review and editing. All authors contributed to the article and approved the submitted version.

## Funding

This study was funded by the China National Social Science Fund Project (20BGL252) and the Independent Innovation Foundation of Wuhan University of Technology (2021-zy-076).

## Conflict of Interest

The authors declare that the research was conducted in the absence of any commercial or financial relationships that could be construed as a potential conflict of interest.

## Publisher's Note

All claims expressed in this article are solely those of the authors and do not necessarily represent those of their affiliated organizations, or those of the publisher, the editors and the reviewers. Any product that may be evaluated in this article, or claim that may be made by its manufacturer, is not guaranteed or endorsed by the publisher.
