# Supplementary material for: Empirical Analysis of the Dynamics of the COVID-19 Epidemic in Urban Embedded Social Networks
Source: Front Public Health. 2022 Jun 2;10:879340. doi: 10.3389/fpubh.2022.879340 (PMC9195001; doi:10.3389/fpubh.2022.879340)

Supplementary Material

1 Supplementary Data

Some information about the cases is listed below. For more information, please visit: http://wjw.nanjing.gov.cn/

Case No.	Sex & Age	Time of definite diagnosis	Occupation	Household location	Activity track
1	F, 40	20 July	Cleaner of Lukou airport	Tongshan Community, Jiangning District	On the morning of July 10, she stayed at home and went to Lukou Tianyu fruit shop at 15:02. On July 11, she work at Lukou Airport. She stayed at home on July 12. From July 13 to July 16, she commuted to Lukou Airport by electric bike every day. On July 17th, she work at Lukou Airport and drove an electric tricycle to Bafang Restaurant for dinner at 17:00. From July 18th to July 19th, She commuted to Lukou Airport by electric bike every day.
2	F, 45	20 July	Cleaner of Lukou airport	Chengong Community, Lukou Subdistrict, Jiangning District	From July 10 to July 17, she commuted to Lukou Airport by electric bike every day. On July 18, she stayed at home in the morning. she went to Lukou Community Health Service Center by electric bike at 17:11 p.m. because of headache, and went home by electric bike at 19:00. On July 19, she went to lukou Airport for work by electric bike at 7:20, went to Lukou Community Health Service Center by electric bike at 15:03, and went home by electric bike at 17:00.
3	F, 51	20 July	Cleaner of Lukou airport	Lukou Subdistrict, Jiangning District	One July 10, she worked in China post air express center, arrive at Maosanmei stationery shop at 12:20 and stay for about 20 minutes. At 8:00 on July 11, she went to Nanjing Lukou Airport to work by electric bike. After 16:00, she accompanied her husband to Tongshan Hospital for vaccination by electric bike. At 17:30, she went home by electric bike. She stayed at home on July 12. On July 13, she went to work at Lukou Airport by electric bike. She stayed at home on July 14. From 8:00 on July 15 to 0:00 on July 16, she commuted to lukou Airport by electric bike. At 8:00 on July 16th, she went to Nanjing Lukou Airport Sub-branch of Bank of China to apply for bank card by motorbike and left at 9:40. 9:40 to Jincheng Style Copy Shop (about 41 meters west of Jincheng College, Nanjing University of Aeronautics and Astronautics).22:30 on July 16 to 10:00 on July 17, she was in China post air express Center.
4	F, 50	20 July	Cleaner of Lukou airport	Shinian Community, Lukou Subdistrict, Jiangning District	On July 16th, 11:00 am, she attended the banquet at Ruijianghong Hotel. In the afternoon, she went shopping at Xiaowu Hardware Store, Nantian Road, and the market near Tongshan Jinken College.
5	F, 48	20 July	Cleaner of Lukou airport	Tongling Community, Lukou Subdistrict, Jiangning District	From July 10 to July 17, she commuted to Lukou Airport every day. On July 18, she went to Tongshan market to buy vegetables at about 9:00. She went to Lantian Road Station at about 12:00 by No. 851 bus, and shopped at Laifeng Road. Then, she took No. 851 bus back to Tongshan market station at about 16:00.

6	F, 48	20 July	Cleaner of Lukou airport	Jiutang Community, Shiqiu Subdistrict, Lishui District	She stayed at home on July 10. From July 11 to July 13, she commuted to Lukou Airport by electric bike every day. She stayed at home on July 14. On July 15, she went to work at Lukou Airport by electric bike. She stayed at home on July 16.
7	F, 38	20 July	Cleaner of Lukou airport	Tongling Community, Lukou Subdistrict, Jiangning District	At about 9:30 on July 11,she with her husband and daughter, went to Tongshan Subway Station in Jiangning District from home. They took subway Line S9 (toward Nanjing South Station), and transfered to subway Line S1 (toward Nanjing South Station) at Xiangyu Road South Station at about 10:16, and get off at Cuipingshan Subway Station in Jiangning District at about 10:40. At 10:48, they went to Baijia Lake Haoyoudao supermarket by online taxi, and then went to Xiaohuniang restaurant on Shengtai West Road for dinner by private car of her relatives At about 13:10, go to Hushan Branch of Jiangning Hospital by online taxi to accompany her husband and daughter to see a doctor. At about 16:00, they went to Cuipingshan Subway station by online taxi. Then, they took subway Line S1, and transfered to Line S9, finally arrived at Tongshan Station at 17:12. She went to Tongshan Community Health Service Center for outpatient treatment at 14:00 on July 16 and returned home at 16:30. She went to the Health Service Center at 9:00 on July 17 and returned home at 11:00.
8	F, 34	21 July	Cabin Cleaner of Lukou airport	Huandunshan Community, Lukou Subdistrict, Jiangning District	After finishing work at Lukou airport at 4:00 on July 10, she went home by motorbike and went shopping at Junjie stewed food shop in Tongshan market at about 10:00. On July 11, she went to work at Lukou airport by electric bike. On July 13, she went to work at Lukou Airport by electric bike. On July 14, she rode an electric bike to Tongshan cold drink wholesale Center and Wangbuliao restaurant near Tongshan market around 17:00, and went shopping in Tongshan jingcheng supermaket at 19:00. From July 15 to 17, she commuted to Lukou airport by electric bike. At about 7:30 on July 18, she went to a bun shop at Tongshan market alone to buy food. At 17:00, she went shopping in Tongshan market and Xiaoyang fruit store.
9	F, 46	21 July	Cabin Cleaner of Lukou airport	Tongshan Community, Lukou Subdistrict, Jiangning District	On July 11, she went to work at Lukou airport by electric bike. From 9:00 to 10:30 on July 12, she went shopping at the Tongshan Haoyouduo supermarket by electric bike. From July 13 to 14, she commuted to lukou Airport by electric bike every day. From 16:10 to 17:25 on July 14, She went to Tongling Road No.249 to buy a mobile phone. On July 15, 17 and 19, she went to work at Lukou airport by electric bike.
10	F, 38	21 July	Driver of cleaning staff in Lukou airport	No.1 airport Community, Lukou Subdistrict, Jiangning District	She went to Baili supermarket during 19:30 to 21:00 every night from July 10 to 11. On July 12, she drove to Lukou airport for work. On July 13, she was at home in the daytime, and took her son to Fili gym at 18:30. She worked as a part-timer at KFC in Lukou airport from 6:00 to 15:00 on July 16, and went to Baili supermarket at 19:00 to 20:30. From 11:15 to 15:00 on July 17, she went to a chess and card room nearby her home, and went to a relatives home for dinner from 17:30 to 18:30, then went to a chess and card room from 18:30 to 20:30. At 9:00 on July 20, she went to Yongxin market by electric bike to buy vegetables. Then, from 11:30 to 16:00, she went to a chess and card room.
11	M, 11	21 July	Student	No.1 airport Community, Lukou Subdistrict, Jiangning District	On July 11, he went to Bailey Square by bike at19:00, and back home at 20:30. At 15:00 on July 12, he bought milk tea at the chicken chop shop downstairs and went to his classmate's house. He activitied with confirmed case 10 on July 13. At 15:00 on July 15, he went to his classmate's home, airport community neighborhood committee, library and other places by bike, and went back home at 17:00. He went to Bailey supermarket by bike at 19:00, and back at 20:30. At 15:00 on July 16th, he went to his classmate's home, airport community neighborhood committee, library and other places by bike, and went back home at 17:00. From 17:30 to 18:30 on July 17, he went to his relatives' house for dinner. He went to Baili Square at 18:40, and back home at 19:00. At 15:00 on July 18, he went to his classmate's

					home, airport community neighborhood committee, library and other places by bike, and came back home at 17:00. He went to to Bailey supermarket by bike at 19:00, and back home at 20:30. On July 19, he went to to Bailey supermarket by bike at 19:00, and back home at 20:30.
12	F, 45	22 July	Cleaner of Lukou airport	Sangyuan Community, Lukou Subdistrict, Jiangning District	From 9:10 to 9:50 on July 11, she shopped at Tongshan market and Suyi supermarket at Tongshan. On July 12, she went to work at Lukou airport by electric bike. She stayed at home on July 13. From July 14 to 18, she commuted to Lukou airirport by electric bike every day. At 9:00 on July 19, she went to the barber shop which is near by Tongshan elementary school at Tongshan community and tongshan market, and then returned home.
13	F, 41	22 July	Cleaner of Lukou airport	Chengang Community, Lukou Subdistrict, Jiangning District	At 10:40 on July 11, she was in shops such as Shaxian Snacks and Tongshan Haoyouduo supermarket in Jincheng Pedestrian Street. From 18:00 to 20:00 on July 12, she stopped at Yangyang cake shop and Wangfa restaurant in Jincheng Pedestrian Street. At 6:40 on July 15, she had dinner at the Tongshan Laoxia noodle restaurant. At 18:05 on July 16, she went shopping in Jincheng pedestrian street and returned home at about 19:30. On July 18, she stopped at Beijing mixed sauce noodle restaurant and Mixuebingcheng cold drink shop in Jincheng pedestrian street. From 8:00 to 8:20 on July 19, she bought vegetables at the Tongshan Shengzhuang market.
14	F, 41	22 July	Cleaner of Lukou airport	Pengfu Community, Lukou Subdistrict, Jiangning District	At 6:00 on July 11, she went to her friend's home in Lishui District for dinner, then returned home. At 17:40 on July 13, she drove herself to the Yuxiaoer restaurant in Haile supermarket in Lishui District and went home at 19:30. At around 18:00 on July 17, she drove to her parents' home in Huandunshan. At about 6:30 on July 19, she went shopping in Tongshan market and picked up the express delivery at Haoyouduo supermarket in Caocun Community.
15	F, 40	22 July	Cabin Cleaner of Lukou airport	Yongxin apartment buildings-Chunlan, Zhongcun Community, Lukou Subdistrict, Jiangning District	At 9:00 on July 11, she went shopping in Gangshan supermarket. On July 12, she went to work at the airport. At 10:00 on July 13, she took bus No. 852 to Nanjing Linjiang High School to get some things (she did not enter the school), and then returned the same way. On July 14, she went to work at the airport. She was at home on July 15.
16	F, 43	22 July	Cabin Cleaner of Lukou airport	Yongxin apartment buildings-Zijing, Zhongcun Community, Lukou Subdistrict, Jiangning District	At 7:39, she stopped near Wenhao Garden, Lukou Street, Jiangning District. From 8:29 to 9:06, she stopped near Fenghuang Community, Jiangning District. From 6:40-9:49 on July 14, she stopped at her husband's workplace (a site near the Committee of Fenghuang Community, Jiangning District). Around 10:09, she rode an electric bike to buy meat in the Xinmao market. At 10:27 and 14:05, she went shopping in the Gangshan supermarket nearby Yongxin apartment buildings-Zijing. From 10:28 to 12:08 July 18, she stopped at her husband's place of work. At 12:00 on July 20, she bought Haiwangxincheng health pharmacy nearby Baili supermarket.
17	F, 44	22 July	Cabin Cleaner of Lukou airport	Xiahuaxi, Shinian Community, Lukou Subdistrict, Jiangning District	From July 10 to 12, she commuted to work at Lukou airport by electric bike every day. At 16:00 on July 13, she drove to Matang Xiejia, Subdistrict and returned at 20:00. From July 14 to 19, I commuted to lukou Airport by electric bike every day.
18	F, 45	22 July	Cleaner of Lukou airport	Shinian Community, Lukou Subdistrict, Jiangning District	On July 11, she went to her son-in-law's house for dinner at 10:00 and returned home at 16:00. On the afternoon of July 15, she went shopping in Tongshan street. At 12:00 on July 17, she bought vegetables in the Tongshan market; at 13:37 she washed her hair in the barber shop near the Tongshan post office; at 19:00 she massaged in the massage parpol at Lukou commodity market. At 16:00 on July 18, she drove herself to her son-in-law's house in Gaochun. Then she drove herself to Liyang Hospital. At

					19:00, she had dinner opposite the hospital and bought fruit in the supermarket nearby. At 11:00 on July 19, she went to the Tongshan market to buy vegetables and had dinner at the gate of the market.
19	F, 45	22 July	Dormitory administrator of Jincheng College	Shinian Community, Lukou Subdistrict, Jiangning District	At 8:00 on July 14, her husband drove her to work and returned home by electric bike at 7:40 on July 15. At about 14:00 on July 15, she met a relative (a confirmed case) at the gate of the community and went home after chatting for about 10 minutes. She stayed at home until July 18. At 7:30 on July 19, she arrived at school on duty.
20	F, 30	22 July	Cleaner of Lukou airport	Baiyunlu Community, Lukou Subdistrict, Jiangning District	At 15:40 on July 10, she rode an electric bike to Lukou market to buy vegetables and then went home. From 7:30 to 13:30 on July 11, she went to work and bought fruit at the Xianguoyi fruit shop near Lukou market on her way home. At 16:40 on July 14, she went shopping at Lukou market by bike. From July 15 to 17, she worked at Lukou airport every day. At 10:30 on July 18, she rode an electric bike to Lukou market to buy vegetables. She went to work on July 19 and visited a relative’s house at 18:00.
21	F, 45	22 July	Cabin Cleaner of Lukou airport	Sangyuan Community, Lukou Subdistrict, Jiangning District	On July 11 to 16, she drove her son to the training class including Chuanwudao budokan, Qizhi training class. At 13:20 on July 17, she sent her son to Minyi dance school, and picked her son up at 17:10 to go home after class, and went to the surrounding supermarket from 18:30 to 19:00. On July 18, she drove her son to Qizhi training class at 7:20. At 8:00 on July 19th, she sent her son to Qizhi training class, then went home. At 16:00, she picked up my son and went home.
22	F, 44	22 July	Cabin Cleaner of Lukou airport	Bailihuafu buildings-No.3, Maoting Community, Jiangning District	At 17:43 on July 10, she went shopping at the Beifangerxian bread shop (No. 6 Kangning Lane, Lukou Street). At 18:31 on July 13, she had dinner at the nearby Dazhuiya restaurant. From 11:11 to 15:00 on July 15, she went to Lukou Community Hospital to get vaccinated. Then she went shopping at Xingmao Supermarket and Xiangsifang marinated food store by electric bike and went home. At 13:30 on July 16, she went shopping in the Star Cat supermarket by scooter and went home. At 14:18 on July 18, she went to xiangsifang marinated food shop and north Erxian steamed bread shop to buy food and go home.
23	F, 19	22 July	Student	Longhu buildings-Wenxin, Jiangning District	At 7:45 on July 10, she walked from her aunt's (confirmed case No.5) home to bozhiwen training center for duty. She did not go out after walking back to her aunt's home at 16:00. From July 12 to 16, she went to Bozhiwen training Center to teach at 7:45 every day, and finished class at 16:00. After class on July 16, she took No. 851 to Jiuzhu Road bus station and walked back to her home in Longhu buildings-Wenxin. She went to Jiuzhu Road bus station at about 6:00 on July 20, take Bus No. 851 to Tongshan Station, get off and walk to Bozhiwen Training Center to teach until 16:00. She went to Tongshan Community Health Service Center after class, and take Bus No. 851 to Tongren hospital fever clinic at 16:20.
24	M, 49	23 July	Mason	Maojiayu Community, Shiqiu Subdistrict,, Lishui District	From 12:00 to 16:00 on July 16, he played mahjong at his neighbor's house in Maojiyu community. On July 19 and 20, he went out to play mahjong with his neighbors.
25	M, 43	23 July	Air logistics company staff	Tongling Community, Lukou Subdistrict, Jiangning District	At 10:48 on July 11, he took an online taxi to baijiahu Haoyouduo supermarket next to the mobile business hall, and then took a private car to Xiaochuniang restaurant at shengtaixi road At about 13:10, he took a taxi to The Hushan Branch of Jiangning Hospital for treatment. At 9:00 on July 20, he went to Tongren Hospital for treatment.

26	F, 51	23 July	Cleaner of Lukou airport	Caocun Community, Lukou Subdistrict, Jiangning District	On the morning of July 17, she went to a neighbor's house. At around 20:00 on July 19, she was shopping at Liangliang store. At about 6:00 on July 20, she rode an electric bike to her mother's home in Jiutang community, Lishui District
27	F, 75	23 July	Peasant	Maocun Community, Lukou Subdistrict, Jiangning District	From July 10 to 14, at around 7:00 am, she walked to the Surunwanjia supermarket on Jinshi Road alone to buy food and then went home. At about 6:00 on July 15, she walked to Lukou Shengzhuang market to buy vegetables and then went home. At around 7:00 on July 16th and 17th, she went shopping at Surunwanjia supermarket on foot. At about 6:00 on July 18, she walked to shengzhuang market to buy vegetables. At about 7:00 on July 19th, she went to Haoyouduo supermarket. Then she went to her relatives' house in Maocun Community. She went to trust-mart at 7:00 on July 20th.
28	F, 31	23 July	Salesman	Cunpingcheng Community, Lukou Subdistrict, Jiangning District	From July 12 to July 21, she shuttled her children to and from Youxing peiyou training institution and went to Fili Gym.
29	M, 40	23 July	Work at airport Community	No.1 airport Community, Lukou Subdistrict, Jiangning District	From July 12 to 16, he visited Baili supermarket and Zuoyongmei marinated food store, ect. On July 17 and 18, he went to Xiangyang Road No.56 and Baili supermarket for activities and shopping. On July 19th, he went to work on foot, then went to the construction site of the airport community, and at 17:00 went to the Xiaozhaor estaurant on Hengxi Street, and had dinner together. He drove to Baili supermarket at 20:30 in the evening, and went home by online taxi at about 22:35.
30	F, 60	23 July	Unemployed	Cunpingcheng Community, Lukou Subdistrict, Jiangning District	From July 16 to 20, she went to the Shushan mahjong parlor frequently.
31	F, 50	23 July	Staff of a company	Zhougang Community, Hushu Subdistrict, Jiangning District	From July 10 to 22, she frequently went to Zhougang chess and card room or nearby friends' homes to play mahjong
32	M, 43	23 July	Driver of Lukou airport	WaicaofangCommunity, Hushu Subdistrict, Jiangning District	From July 10 to 21, he played majhong in Xiangyang community after work. At 17:13 on July 10, he went to Lukou Community Hospital to deliver things. At 15:28 on July 14, he drove to Tongren Hospital to accompany his wife to see a doctor. On July 21, he drove to Sanqitang pharmacy to buy medicine.
33	M, 21	23 July	Unemployed	Shinian Community, Lukou Subdistrict, Jiangning District	At about 10:00 on July 11, he drove to his relatives' home in Yaxi subdistrict, Gaochun District for dinner. At 16:00 on July 18, he drove to Gaochun with confirmed case No.18 and No.34, then went to Liyang People's Hospital to visit the patient and returned home at 20:00. At 11:00 on July 19, he went to the Tongshan market with confirmed case No.8 and No.34, ate at the gate of the market.
34	M, 46	23 July	Taxi driver	Shinian Community, Lukou Subdistrict, Jiangning District	At about 10:00 on July 11, he drove to his relatives' home in Yaxi subdistrict, Gaochun District for dinner. At 16:00 on July 18, he drove to Gaochun with confirmed case No.18 and No.33, then went to Liyang People's Hospital to visit the patient and returned home at 20:00. At 11:00 on July 19, he went to the Tongshan market with confirmed case No.18 and No.33, ate at the gate of the market.
35	F, 67	23 July	Peasant	Sangyuan Community, Lukou Subdistrict, Jiangning District	From July 10 to July 21, she worked at home daily. At 13:00 on July 14, she rode an electric bike to Tongshan Health Service Center for vaccination, then returned home.

36	M, 53	24 July	Cleaner of Lukou airport	Maoting Community, Lukou Subdistrict, Jiangning District	From July 10 to July 23, he commuted to Lukou airport by electric bike every day. At 22:43 on July 15, he went shopping at Domai supermarket.
37	M, 27	24 July	Airline ground crew	Siyuan Community, Nanyuan Subdistrict, Jianye District	The working hours of this case were not fixed, and there were work records on July 12, 14, 16 and 19. The mode of transportation was to drive a car to and from lukou Airport where he worked and his current address. After work, he mainly stayed at home and seldom went out.
38	F, 46	25 July	Tradesman of building materials	Yaxi Subdistrict, Gaochun District	At about 18:00 on July 18, she, confirmed case No.18 and No.34 went to Liyang People's Hospital by private car. At 5:30 on July 19, she went to Dingbu market to buy some vegetables and took her private car to Liyang People's Hospital at 8:00.
39	F, 37	25 July	Unemployed	Zhenzhubei Road, Yongyang Subdistrict, Lishui District	At 16:30 on July 13, she took a taxi (SuAD39458) to Lukou airport, took flight HO1693 from Lukou airport at 17:25, and returned to Nanjing by flight PL6259 on July 17. After arriving at dawn on July 18, she took a bus back to Lishui and went to live in Xiangzhangyuan residence. At 9:30 on July 19, she went to Baiwoyouxian supermarket to buy food.
40	F, 40	25 July	Cleaner of Lukou airport	Biguiyuan, Shiqiu Subdistrict, Lishui District	On July 10, she rode an electric scooter to Lukou airport. On July 11, she went shopping at Lijialiang bread shop at 6:20 and Hanwen department Store at 15:40. At 20:50 on July 13, she went to Tongshan Gas station to refuel.
41	F, 39	25 July	Cabin Cleaner of Lukou airport	Shinian Community, Lukou Subdistrict, Jiangning District	At 6:37 on July 10, she bought breakfast at the Zhengquanwei store nearby Jincheng College. She went to her parents' home (Xihuanxi, Shinian Community, Jiangning District) at 10:00 on July 11 and return home at 19:00. On July 13, she went to my parents' house at 10:00. At about 19:00, she went to Yanian pharmacy to buy medicine and returned home. She went shopping at Tongshan Market at 7:14 on July 14. From 8:30 to 11:00 on July 15, she went to tongshan Health Center for treatment, then went to her parents' home and returned home at 19:00.
42	F, 43	25 July	Cleaner of Lukou airport	Tongshan Community, Lukou Subdistrict, Jiangning District	At 10:00 on July 10, she rode an electric bike to the Suguo supermarket for shopping. From July 13 to 15, she commuted to work at Lukou airport by electric bike. At 10:30 on July 16, she went shopping at Tongshan cold drinks wholesale center.
43	M, 42	25 July	Auxiliary polic of Lukou airport	Huandunshan, Caocun Community, Lukou Subdistrict, Jiangning District	At 7:50 on July 13, he went shopping at Laoliu fruit Store. At 14:5 on July 16, 8, he went shopping in LaoLiu Fruit Store and Liangliang Store. At 12:36 on July 17, he went to Tongling Road Zhengtian training institution by motorbike.
44	F, 42	25 July	Cabin Cleaner of Lukou airport	Xiecun Community, Lukou Subdistrict, Jiangning District	At 11:30 on July 12, 17 and 19, she went to tongling Mahjong house, and at 16:00 she went to Tongshan Vegetable market to buy vegetables and went home. She had dinner at Lishui Huawei Hotel from 9:00 to 16:00 on July 14. At 21:00, she went to eat at xiaojungan restaurant in Jincheng College pedestrian Street by car and went home at 22:00.
45	F, 43	25 July	Cabin Cleaner of Lukou airport	Shinian Community, Lkou Subdistrict, Jianye District	At 9:45 on July 19, she went to Yongxin apratment buildings, Jiangning District by electric bike. At 14:00, she rode to the Lukou Market on Blue Sky Road.
46	F, 43	25 July	Cleaner of Lukou airport	Yongxin apratment buildings-Zijing, Lukou	On the morning of July 15 and 16, she hosted a dinner at Ruijianghong Hotel. On the evening of July 17, she ate Shiweixuan restaurant at zhengyang Road.

				Subdistrict, Jianye District	
47	M, 52	25 July	Plumber of Lukou airport	Zhetang Subdistrict, Lishui District	From July 10 to 19, he cycled to and from the airport.On the afternoon of July 21, he went to the cainiao post station in the residential area on his way home from the airport.
48	F, 41	25 July	Cleaner of Lukou airport	Xiaopeng Community, Lukou Subdistrict, Jiangning District	On July 16th, she went to Tongren hospital and then to Haoyouduo supermarket on Baiyun Road. On July 19, she went to work at the airport by bike and went to Shinian stewed food shop after 14:30.
49	F, 48	25 July	Cleaner of Lukou airport	Xiecun Community, Lukou Subdistrict, Jiangning District	On July 10, she ate at the restaurant opposite Suguo Supermarket at 12:00 and went shopping at XiaoLiang electric appliance store at 16:00. She went to work at the airport on July 11th. She stayed at home on July 12. She went to work at the airport from July 13 to 17.
50	F, 50	25 July	Unemployed	Waicaofang Community, Lukou Subdistrict, Jiangning District	On July 12, she visited Ruyi Lake in Jiangning district. On July 16, she went to Tongren hospital, and then she went to Tongren Pharmacy to buy medicine and Lanlan Road vegetable market to buy food. She was identified as a close contact on July 21.
51	F, 18	25 July	Student	Tongshan Community, Lukou Subdistrict, Jiangning District	Between 12 and 20 July, she walked twice to visit relatives nearby home where she contacted with a confirmed cases.
52	F, 48	25 July	Cleaner of Lukou airport	No.1 airport Community, Lukou Subdistrict, Jiangning District	On July 11, she went shopping at the Wing Hin Road vegetable market. On July 14, she went to shengzhuang Vegetable Market to buy some vegetables. She was identified as a close contact on July 20.
53	F, 72	25 July	Peasant	Shinian Community, Lukou Subdistrict, Jiangning District	From July 10 to 20, she went to shangwanxi to activity several times, the rest of the time at home.
54	M, 30	25 July	Airline ground crew	Qinhuai District	From July 10 to 20, he drove himself to lukou Airport for work. At 8:50 on July 11, he had breakfast at 1 Guanghua East Street near his home, then he went for a walk in Yueya Lake Park.
55	F, 60	25 July	College cleaner	Xiecun Community, Lukou Subdistrict, Jiangning District	She worked at the college from July 8 to 20. She stayed at home on July 11, 21 and 22. At 9:15 on July 18, she went to her daughter's house for dinner and was driven home by her son-in-law at 13:00.
56	F, 52	25 July	Cleaner of Lukou airport	Tongshan Community, Lukou Subdistrict, Jiangning District	From July 9 to 19, she occasionally went to Tongling Mahjong parlour. At 15:30 on July 14, she drove to Huangniu hotpot restaurant with her friend (confirmed COVID-19 case) in Shiqiu Subdistrict.
57	F, 67	25 July	Sanitation worker	Baiyunlu Community, Lukou Subdistrict, Jiangning District	From 18:30 to 20:40 on July 17, she played mahjong in Shushan Mahjong Parlor.
58	M, 33	25 July	Lukou airport staff	Shenglicun Community, Xiaolinwei Subdistrict, Xuanwu District	He worked from July 10 to 12. At noon on July 12, he had dinner at the Xinshiji Hotel. At 10:50 on July 17, he drove himself to visit his mother-in-law's house.

59	F, 56	25 July	Retired	Xiecun Community, Lukou Subdistrict, Jiangning District	From July 12 to 19, she played mahjong in Tongling mahjong room many times.
60	F, 53	25 July	College dormitory administrator	Xiecun Community, Lukou Subdistrict, Jiangning District	On the afternoon of July 19, she was playing mahjong in tongling Mahjong Parlor.
61	F, 47	25 July	Cleaner of Lukou airport	Tongshan Community, Lukou Subdistrict, Jiangning District	On the afternoon of July 19, she was playing mahjong in tongling Mahjong Parlor.
62	F, 41	25 July	College dormitory administrator	Shinian Community, Lukou Subdistrict, Jiangning District	She worked at the college from July 12 to 20.
63	M, 40	25 July	Truck driver	Yongxin apartment buildings-Chuanlan, Yongxin Community, Lukou Subdistrict, Jiangning District	After July 13, he had been to Yonghui supermarket, Lantianlu vegetable market, Lukou Haoyouduo supermarket, etc.
64	M, 65	25 July	Unemployed	Yongxin Community, Lukou Subdistrict, Jiangning District	From July 11 to 19, he spent most of his time at home, occasionally visiting the market on Blue Sky Road, and theShushan mahjong parlour.
65	F, 41	25 July	Cleaner of Lukou airport	Yongxin apartment buildings-Haitang, Yongxin Community, Lukou Subdistrict, Jiangning District	She worked at the college from July 8 to 20. She stayed at home on July 11, 21 and 22. At 9:15 on July 18, she went to her daughter's house for dinner and was driven home by her son-in-law at 13:00.
66	F, 63	25 July	Unemployed	Shinian Community, Lukou Subdistrict, Jiangning District	She went to Yongxin apartment buildings every morning from July 11 to 20. From 9:15 to 15:30 on July 12, she took an online taxi to send her children to class from Yongxin. On July 15, she went to Chenhong swimming gym by private car. 19:00 July 19, went to neighbor's house to play mahjong in which she contacted with confirmed case.
67	F, 43	25 July	Cleaner of Lukou airport	Hengfeng Road No.20, Tongshan Subdistrict, Jiangning District	From July 10 to 17, she worked at the airport most of the time. At 11:30 on July 18, she rode to Jingken College to pick up a delivery. At 9:00 on July 20, she took an online taxi to Wuzhou Hospital.
68	M, 44	25 July	Stevedore of Lukou airport	Xiaopengcun Community, Lukou Subdistrict, Jiangning District	On the morning of July 12, he went shopping at Tongshan market and Cold drink Wholesale Center nearby the east gate of Tongshan vegetable market. On July 14th, he went to Tongren Hospital at 8:00 and had dinner at Tongshan market at 11:00. In the morning of July 16, we had dinner at Tongshan vegetable market. On the morning of July 18, he went to buy vegetables at Tongshan market.
69	F, 44	25 July	College dormitory administrator	Shinian Community, Lukou Subdistrict, Jiangning District	From July 11 to 20, she worked at the school most of the time.

70	M, 44	25 July	Airline ground crew	Xiecun Community, Lukou Subdistrict, Jiangning District	From July 9 to 18, he was at the airport, and on July 11, 13, 14 and 17, he worked as a part-time manager at the Tongshan market. At 9:00 on July 19, he went to the Tongshan market for dinner, at 19:00 he went to the Tongshan Community Health Service Center, and at 21:00 he drove to the Xiaojungan restaurant on the high street of Jincheng College for dinner.
71	M, 38	25 July	Unemployed	Xiecun Community, Lukou Subdistrict, Jiangning District	He worked at the main window shop from July 9 to 17, sometimes going out to make house calls. On July 17, he installed window screens at Biguiyuan in Lishui District.
72	M, 24	25 July	Airline ground crew	Honglihuayuan, Lishui District	From July 10 to 19, he visited Xipuhongyuan Museum and Lishui District Hospital of Traditional Chinese Medicine, etc.
73	F, 59	25 July	Unemployed	Maigaoqiao Subdistrict, Xixia District	On July 20, she hosted her brother who come from the airport.
74	M, 9	25 July	Student	Cuipingchen Community, Lukou Subdistrict, Jiangning District	From July 11 to 21, he spent most of his time in youxingpeiyou training class, supermarkets and parks near his home. He was identified as a close contact on July 22.
75	M, 3	25 July	Infant	Cuipingchen Community, Lukou Subdistrict, Jiangning District	He acted with his family member who is a confirm cases.
76	F, 53	26 July	Unemployed	Shangqiao Community, Hushu Subdistrict, Jiangning District	On the evening of July 17, 18 and 20, she went to Zhougang chess and card room.
77	F, 50	26 July	Unemployed	Xiecun Community, Lukou Subdistrict, Jiangning District	On July 18, 19 and 20, she played mahjong in Tongling Mahjong Parlour and contacted with confirmed cases. She was identified as a close contact on July 22.
78	M, 52	26 July	Massagist	Qunli Community, Lukou Subdistrict, Jiangning District	From July 10 to 20, he worked in the massage parlor at Lukou commodity market.
79	M, 55	26 July	Airline ground crew	Shinian Community, Lukou Subdistrict, Jiangning District	From July 10 to 19, he commuted to the airport for work.
80	M, 40	26 July	Cleaner of Lukou airport	Caocun Community, Lukou Subdistrict, Jiangning District	From July 11 to 20, he commuted to the airport for work.
81	F, 51	26 July	Cleaner of Lukou airport	Yongxin apartment buildings-Zijing, Lukou Subdistrict, Jiangning District	From July 10 to 22, she worked at the airport most of the time. At 16:30 on July 15, she cycled to her daughter's home in Shanghuanxi, Shinian Community for dinnerr.

82	F, 45	26 July	Cleaner of Lukou airport	Mapu Community, Lukou Subdistrict, Jiangning District	From July 11 to 21, she worked at the airport most of the time. She went to Yongxin apartment buildings-Zijing at 9:00 on July 17.
83	F, 63	26 July	Retired	Waicaofang Community, Lukou Subdistrict, Jiangning District	Between 11:10 and 13:00 on 17 July, she was in a mahjong parlor near her home where she contacted with confirmed cases. On the morning of July 20, she went to her daughter's house for dinner. At 7:00 on July 21, she went shopping at the Haoyouduo supermarket on Wenxuan road. At 13:00 she went to a friend's house on Zhengyang road.
84	F, 48	26 July	Cleaner of Lukou airport	Xiaopeng Community, Lukou Subdistrict, Jiangning District	From July 11 to 21, she worked at the airport most of the time. From 8:00 to 9:00 on July 15, she went to the Tongshan market to buy vegetables.
85	F, 42	26 July	Cleaner of Lukou airport	Yongxin apartment buildings-Zijing, Lukou Subdistrict, Jiangning District	From July 11 to 21, she worked at the airport most of the time. From 8:30 to 9:30 on July 12 and 18, she went to the vegetable market at the north gate of Yongxin apartments to buy food.
86	F, 45	26 July	Cleaner of Lukou airport	Caocun Community, Lukou Subdistrict, Jiangning District	On July 15 and 16, she attended the banquet at Ruijianghong Hotel.
87	F, 44	26 July	Cleaner of Lukou airport	Xiecun Community, Lukou Subdistrict, Jiangning District	From July 10 to 20, she worked at the airport most of the time. On July 19, she went to Tongling Market to buy vegetables at 10:00 a.m. and had dinner with her family at Tongling road No.145 restaurant at 18:00 p.m.
88	F, 47	26 July	Cleaner of Lukou airport	Xiaopeng Community, Lukou Subdistrict, Jiangning District	From July 10 to 20, she worked at the airport most of the time.
89	M, 37	26 July	Taxi driver	Daqiao south road No.10, Yijiangmen Subdistrict, Gulou District	Due to occupational reasons, the trajectory of the case was relatively complicated, and the trip involved all districts in Nanjing except Liuhe district, Lishui District and Gaochun District, and there were several trips to Lukou Airport area.
90	F, 49	26 July	Cleaner of Lukou airport	No.1 airport Community, Lukou Subdistrict, Jiangning District	From July 10 to 20, she worked at the airport most of the time. At 18:00 on July 12, she went to the fruit shop at the gate of the Liwaicheng to buy fruit. At 9:00 on July 19, she went to the vegetable market at the north gate of Yongxing apartment.
91	M, 46	26 July	Cleaner of Lukou airport	Gengfang Community, Lukou Subdistrict, Jiangning District	From July 13 to 18, he worked at the airport most of the time. On July 15, he went to Tongshan vegetable market.
92	F, 49	26 July	Cleaner of Lukou airport	Tongshan Community, Lukou Subdistrict, Jiangning District	On July 11 and 14, she went to the Tongsha market to buy vegetables. From 16:00 to 18:00 on July 19, she went to she went to clean the house of a resident who live at Liwaicheng community.

93	F, 48	26 July	Cleaner of Lukou airport	Xucheng Community, Hengxi Subdistrict, Jiangning District	From July 11 to 20, she worked at the airport most of the time
94	F, 42	26 July	Cleaner of Lukou airport	Chengang Community, Lukou Subdistrict, Jiangning District	From July 10 to 15, she cycled to and from work. At 8:00 on July 16, she took her son to Tongshan Community hospital by bike, and then went to the drugstore opposite the Suguo Supermarket to buy medicine. From July 17 to 19, she cycled to and from work.
95	M, 48	26 July	Tradesman of building materials	Yaxi Subdistrict, Gaochun District	He acted mainly around his own store. On July 23, he was identified as a close contact.
96	M, 30	26 July	Restaurant staff of Lukou airport	Shinian Community, Lukou Subdistrict, Jiangning District	On July 10, he went shopping in Gangshan Supermarket. He went to work on July 11. On July 12, he went shopping at tongshan market and Suguo supermarket. After work on July 13, he went shopping at Haoyouduo supermarket. From July 15 to 19, he cycled to and from work.
97	M, 27	26 July	Camerisrt	Dongshan Subdistrict, Jiangning District	On July 16 and 19, he took a flight at Lukkou Airport.
98	M, 54	26 July	Bussinessman	Moling Subdistrict, Jiangning District	He usually works at home. Between July 12 and 17, he often walked around the neighborhood.
99	F, 17	26 July	Student	Yongxin apartment buildings-Haitang, Lukou Subdistrict, Jiangning District	She attended school from July 10 to 13. On July 14, she went shopping at a stationery store near Gangshan supermarket. On July 16, she went shopping with her mother near Lukou Street. On the evening of July 17, she went to the express delivery point at the gate of the community to get the express. On the evening of July 18, she went shopping near Lukou Street and Maoting Road.
100	F, 26	26 July	Cabin cleaner of Lukou airport	Lukou Street, Lukou Subdistrict, Jiangning District	From July 10 to 17, she worked at the airport most of the time. From 18:00 to 19:00 on July 12, she drove herself to Ruyi Lake. She took an online taxi to Tongren Hospital at 7:15 on July 16. 10:15 She rode an electric bike to the Lantian road market to buy food.
101	F, 55	26 July	Cabin cleaner of Lukou airport	Zhougang Community, Hushu Subdistrict, Jiangning District	From July 10 to 19, she worked at the airport most of the time. At 9:27 on July 13, she went to Zhougang market to buy vegetables.
102	M, 68	26 July	Unemployed	Waicaofang Community, Lukou Subdistrict, Jiangning District	He stayed at home and did not go out from July 10 to 20.
103	F, 46	26 July	Cleaner of Lukou airport	Baiyunlu Community, Lukou Subdistrict, Jiangning District	On July 10, 12, 15 and 16, she went shopping in Surunwanjia supermarket and went to her parents' home in Yongxin apartmen buildings-Meigui after dinner. At 8:00 on July 18, she went to Lukou Community Health Service Center for treatment. At 9:22, she bought food in Shengzhuang vegetable market.
104	F, 41	26 July	Cleaner of Lukou airport	Shinian Community, Lukou Subdistrict, Jiangning District	On the morning of July 12, she went to the Tongshan market to buy vegetables. On the afternoon of July 14, she went shopping at Tongshan market and Caocun marinated restaurant. At 14:00, she went to Jiutang community, Shiqiu Subdistrict, Lishhui District. At 16:00 on July 18, she went shopping at Qiangsheng grain Store and bought vegetables at 16:30 at Tongshan market.

105	F, 48	26 July	Cleaner of Lukou airport	Shinian Community, Lukou Subdistrict, Jiangning District	From July 10 to 19, she worked at the airport most of the time. At about 9:00 on July 13, she went shopping at Tongshan market. At around 8:00 on July 20, she accompanied her daughter to gulou Hospital by an online taxi
106	F, 44	26 July	Cabin cleaner of Lukou airport	Tongshan Community, Lukou Subdistrict, Jiangning District	From July 10 to 19, she worked at the airport most of the time. At 9:50 on July 11, she went to my father's house at Shiqiu Subdistrict. At 6:00 on July 20, she went to Shengzhuang vegetable market to buy food.
107	M, 37	27 July	Stevedores of Lukou airport	Tongshan Community, Lukou Subdistrict, Jiangning District	From July 10 to 19, he worked at the airport most of the time. On July 12, he went to the Tongshan Haoyouduo supermarket. Around 6:40 on July 19, he was eating breakfast across the street from the Copper Hill Community Health Service.
108	F, 49	27 July	Cleaner of Lukou airport	Lukou Subdistrict, Jiangning District	From July 10 to 19, she worked at the airport most of the time. At 10:00 on July 17, she went to the Tongshan Community Health Service Center. At 14:41 on July 18, she went to Gangshan supermarket,and at 15:20, she went to the Tongshan Community Health Service Center. At 5:30 on July 20, she went shopping at Tongshan vegetable market.
109	F, 46	27 July	Cabin cleaner of Lukou airport	Gengfang Community, Lukou Subdistrict, Jiangning District	From July 10 to 20, she worked at the airport most of the time.
110	M, 43	27 July	Airline ground crew	Bailihuafu, Lukou Subdistrict, Jiangning District	On July 17, he drove himself to and from Lukou Airport. At 19:00 after work, he went to Lukou Community Health Service Center for medical treatment. At around 9:00 on July 18, he went to Shijihualian supermarket ait Baili square and stayed for about 30 minutes.
111	M, 44	27 July	Airline ground crew	Maoting Community, Lukou Subdistrict, Jiangning District	At 3:00 on July 10, he drove home from work and went to his relatives' house at 11:00. On July 11, he drove himself back and forth to work at Lukou airport. At 9:20 on July 12, he went to Shengzhuang vegetable market to buy vegetables. He drove himself to work at the airport at 8:00 on July 13. At 10:20 on July 16, he went to Shengzhuang vegetable market to buy vegetables.
112	M, 51	27 July	Construction supervisor of Lukou airport	Yongxin apartment buildings-Mudan, Lukou Subdistrict, Jiangning District	From July 10 to 19, he worked at the airport most of the time. Sometimes he went to market at the north gate of Yongxin apartment buildings.
113	M, 52	27 July	College dormitory administrator	Shinian Community, Lukou Subdistrict, Jiangning District	She went to work at school from July 13 to 17, and went shopping at Tongshan Suguo supermarket at noon on July 16. She went to work at the school, and contacted with confirmed cases on 19 July.
114	M, 27	27 July	Airline maintenance who went to Nanjing on business	–	From July 10 to July 25, his main activities were in Lukou area of Jiangning District and Lukou airport in Nanjing.
115	F, 34	27 July	Staff of a company	Baiyunlu Community, Lukou Subdistrict, Jiangning District	From July 12 to 20, she went to the card room near Maoting Road for many times, and also went to Baiyun Road and Baili Square to buy drink.

116	F, 39	27 July	Cleaner of Lukou airport	Shinian Community, Lukou Subdistrict, Jiangning District	From July 10 to 19, she worked at the airport most of the time.
117	M, 34	27 July	Cleaner of Lukou airport	Tongshan Community, Lukou Subdistrict, Jiangning District	He worked at the airport from July 12 to 18. On the evening of July 19, he went shopping at Yongzheng supermarket
118	F, 46	27 July	Cleaner of Lukou airport	Sangyuan Community, Lukou Subdistrict, Jiangning District	From July 10 to 19, she worked at the airport most of the time. On July 14, 18, she went to Huangniurou restaurant at Shiqiu Road, Lishui District for dinner.
119	F, 45	27 July	Cleaner of Lukou airport	Chengang Community, Lukou Subdistrict, Jiangning District	On the evening of July 15, she went to Ruijiang Hotel for dinner. At noon on July 16, she went to Ruijiang Hotel for dinner.
120	F, 53	27 July	Cleaner of Lukou airport	Tongshan Community, Lukou Subdistrict, Jiangning District	From July 10 to 19, she worked at the airport most of the time. On July 19, she went to Tongshan Market, Tongling Road Suguo supermarket and Tongren Hospital
121	F, 51	27 July	Tradesman of marinate food shop	Shinian Community, Lukou Subdistrict, Jiangning District	From July 12 to 19, she usually buy vegetables at the Tongshan market at about 6:00 every day, and then went to Shinian marinate food shop for processing and operation until about 18:00.
122	F, 50	27 July	Cleaner of Lukou airport	Xiaotaojia Community, Zhetang Subdistrict, Lishui District	She stayed at home from July 10 to 20 except for working at the airport.
123	M, 69	27 July	Peasant	Sangyuan Community, Lukou Subdistrict, Jiangning District	On July 11, he attended a banquet at Wendemu Hotel in Lukou Subdistrict, Jiangning District. He was indentified as a close contact on July 22.
124	F, 49	27 July	Cleaner of Lukou airport	Chenggong Community, Lukou Subdistrict, Jiangning District	She stayed at home from July 10 to 19 except for working at the airport.
125	F, 36	27 July	Staff of Community health service center	Yongxin apartment buildings-Haitang, Lukou Subdistrict, Jiangning District	She stayed at home from July 10 to 19 except for working at the airport.
126	M, 8 months	27 July	Infant	Zhenzhubei Community, Yongyang Subdistrict, Lishui District	He is the son of the confirmed case and has the same activity track as the confirmed case.
127	M, 8	27 July	Student	Zhenzhubei Community, Yongyang Subdistrict, Lishui District	He is the son of the confirmed case and has the same activity track as the confirmed case.

128	F, 49	27 July	Cleaner of Lukou airport	Maoting community, Lukou Subdistrict, Jiangning District	She stayed at home from July 12 to 19 except for working at the airport.
129	M, 76	27 July	Retired	Xiecun community, Lukou Subdistrict, Jiangning District	From 10:00 to 15:00 on July 12 to 19, he went out to shop and play cards, and stayed at home the rest of the time. On July 22, he was indetified as a close contact.
130	M ,43	27 July	Pilot of Lukou airport	Sangyuan community, Lukou Subdistrict, Jiangning District	From July 12 to 22, he worked at the airport most of the time. On July 15, he drove to Wushan Town, Lishui District to pick up colleagues and go home together. On July 24, he was indetified as a close contact.
131	F, 80	27 July	Retired	Litang community, Lukou Subdistrict, Jiangning District	She was at home from July 11 to 25.
132	M, 37	27 July	Cleaner of Lukou airport	Zhougang community, Hushu Subdistrict, Jiangning District	From July 10 to 20, he worked at the airport most of the time.
133	F, 40	27 July	Staff of Lukou airport	Shazhou Subdistrict, Jianye District	She worked at the airport on July 13. She went to her parents' house on July 14. She took a taxi to the airport for her flight around 15:00 on July 15. On July 19, she took a flight back to Nanjing and took a taxi home. She drove to work on July 20.
134	F, 26	27 July	Piano teacher	Waicaofang community, Hushu Subdistrict, Jiangning District	From July 11 to 19, she worked at the airport most of the time. She went Lukou market, Haoyouduo supermarket and Lantianlu store, etc.
135	M, 37	27 July	Staff of a company	Zhenzhubei Community, Yongyang Subdistrict, Lishui District	On July 13, he went to the airport and flew back on July 17. At 7:00 on July 20, he went shopping in baiwoyouxian supermarket near his home.
136	F, 57	27 July	Unemployed	Baiyunlu community, Lukou Subdistrict, Jiangning District	She usually went to the Shushan card parlor. She went shopping at FXianguoyigou supermarket at 9:00 on July 20.
137	M, 31	27 July	Stevedores of Lukou airport	No.1 airport Community, Lukou Subdistrict, Jiangning District	From July 11 to 19, he worked at the airport most of the time. He was identified as a close contatc on July 23.
138	F, 11	27 July	Student	Zhenzhubei Community, Yongyang Subdistrict, Lishui District	From July 13 to 18, she stayed at her grandmother's house. She took an online taxi home at 15:00 on July 19. She was identified as a close contatc on July 24.

139	F, 2	27 July	Infant	Shinian community, Lukou Subdistrict, Jiangning District	She is the granddaughter of the confirmed case and has the same travel schedule as the confirmed case.
140	F, 52	27 July	Staff of a water plant	Waicaofang community, Lukou Subdistrict, Jiangning District	From 18:00 to 22:00 on July 18, she was in the mahjong hall opposite the Haoyouduo supermarket. On July 21, she went shopping in Shengzhuang vegetable market and Haoyouduo supermarket.
141	M, 55	27 July	Cleaner of Lukou airport	Yongxin community, Lukou Subdistrict, Jiangning District	At noon on July 16th, he attended a banquet in Ruijianghong Hotel. He went shopping in Suguo supermarket on July 22.
142	M, 35	27 July	Photo studio owner	Liwaicheng community, Lukou Subdistrict, Jiangning District	On July 12, he went to work at Baihua photo studio. On July 22, he went to Haoyouduo supermarket at Wenxuan road.
143	M, 51	27 July	Renovation worker	Baiyunlu community, Lukou Subdistrict, Jiangning District	He was at the mahjong parlor from 11:00 to 15:00 on the 17, and contacted with confirmed cases. At noon on July 21, he rode to Yongxin apartment buildings-Haitang for dinner. At 9:00 on July 24th, he went to the vegetable market at th north gate of Yongxin apartment buildings.
144	M, 30	27 July	Airline ground crew	Yongxin apartment buildings-Haitang, Lukou Subdistrict, Jiangning District	From July 13 to 20, he worked at the airport most of the time. He went shopping at Gangshan supermarket at 18:00 on July 21
145	F, 9	27 July	Student	Cuipingcheng community, Lukou Subdistrict, Jiangning District	She went to her classmate's house to play on July 19, and contacted with confirmed cases. She walked to her grandfather's house at 19:00 on July 21.
146	F, 31	27 July	Miscwork	Cuipingcheng community, Lukou Subdistrict, Jiangning District	She is the mother of the confirmed case and had the same travel schedule as the confirmed case.
147	F, 47	27 July	Cleaner of Lukou airport	Yongxin apartment buildings-Mudan, Lukou Subdistrict, Jiangning District	From July 12 to 20, she worked at the airport most of the time. On July 20, she went to the cold drink wholesale store on Lantian Road
148	F, 13	27 July	Student	Liwaicheng Community, Lukou Subdistrict, Jiangning District	She went to the Baihuai photo studio on July 14. At 13:00 on July 17, she watched a movie in Baili supermarket, and at 18:00 she went to swim in Chenhong swimming club. On July 21, she went to Haoyouduo supermarket at baiyun Road. On July 23, she went to a friend's house for dinner with her family.
149	F, 49	27 July	Staff of Lukou airport	Zhongyangmen Subdistrict, Gulou District	From July 13 to 21, she worked at the airport most of the time.

150	M, 54	27 July	Staff of Lukou airport	Tiexinqiao Subdistrict, Yuhuatai District	From July 14 to 21, she worked at the airport most of the time.
151	F, 24	27 July	Unemployed	Yongxin apartment buildings-Haitang, Lukou Subdistrict, Jiangning District	She was shopping at Gangshan supermarket on July 22. On July 24, she went to Xianguoyigou supermarket.
152	M, 31	27 July	Researcher	Mochouhu Subdistrict, Jianye District	On July 13, he took a flight at Lukou Airport. On July 17, he returned to the airport.
153	M, 54	27 July	Postman of Lukou airport	Xiecun Community, Lukou Subdistrict, Jiangning District	From July 13 to 20, he worked at the airport most of the time. On July 15, he went to milk tea shop at Jincheng college to buy milk tea.
154	F, 33	28 July	Kingergarener	Qianjiacun Community, Hushu Subdistrict, Jiangning District	On July 11, 12, 17 and 20, he went to play cards at friends' houses in the Gaoqiao community. At 18:50 on July 18, he went to a friend's house near Zhougang Community Health Service Center.
155	M, 60	28 July	Unemployed	Fengxiangxincheng, Yuhuatai District	From July 10 to 20, he spent most of his time near his home. During this period, he drove to lukkou Wing Hing community to meet friends twice.
156	F, 68	28 July	Rtired	Yongxin apartment buildings-Haitang, Lukou Subdistrict, Jiangning District	She often go to the Baolan fur factory to deliver vegetables to her daughter, and occasionally plays mahjong at the Shushan chess and card parlor
157	M, 67	28 July	Peasant	Waicaofang Community, Lukou Subdistrict, Jiangning District	At 9:00 on July 21, he rode to the Haoyouduo supermarket on Wenxuan Road. At 6:40 on July 22, he played chess with a friend. He was indentified as a close contatc on July 25.
158	F, 56	28 July	Unemployed	Chenggong Community, Lukou Subdistrict, Jiangning District	From July 10 to 20, he went to play mahjongg several times at the Yongxin community chess and card room. During this period, he contacted with confirmed cases.
159	M, 51	28 July	Plumber of Lukou airport	Zhangqiao Community, Lukou Subdistrict, Jiangning District	From July 10 to 20, he spent most of his time to work at the airport. At 14:30 on July 22, he went shopping in the Xingmao supermarket.
160	F, 42	28 July	Cleaner of Lukou airport	Chengang Community, Lukou Subdistrict, Jiangning District	From July 12 to 19, she worked at the airport most of the time. At 6:55 on July 16, she had a meal at Lanzhou ramen restaurant opposite Tongshan market with her colleagues who is a confirmed case.
161	M, 70	28 July	Retired	Xiaopeng Community, Lukou Subdistrict, Jiangning District	On the morning of July 18, he went to Copper Hill Community Hospital. Between July 19 and July 25, he went to a neighbor's house several times to play cards. He was idenified as a close contatc on July 25.

162	M, 27	28 July	Planner	Chalu Community, Dongshan Subdistrict, Jiangning District	He visited Nanjing high-speed railway station on July 21, 23, 24 and 25. At 21:00 on July 27, he drove himself to Xihua Building.
163	F, 67	28 July	Peasant	Waicaofang Community, Lukou Subdistrict, Jiangning District	She was playing mahjong at home from 12:00 to 14:00 on 18 July when a confirmed case was involved. At 9:00 on July 21, she went shopping at Haoyouduo supermarket on Wenxuan road.
164	F, 75	28 July	Peasant	Waicaofang Community, Lukou Subdistrict, Jiangning District	In the morning of July 14, she had a meal with her son who is a driver worked at Lukou airport, and in the afternoon she went to Shushan chess and card room. On July 23, she went to Haoyouduo supermarket on Baiyun road.
165	M, 56	28 July	Stevedore of a logisitic company	Shinian Community, Lukou Subdistrict, Jiangning District	As he lives in a high-risk area, he has been quarantined at home since July 21.
166	F, 58	28 July	Doctor	Shinian Community, Lukou Subdistrict, Jiangning District	She commuted to the hospital daily from 13 to 20 July. On July 18, she saw a villager who was later diagnosed.
167	F, 67	28 July	Peasant	Waicaofang Community, Lukou Subdistrict, Jiangning District	She had unprotected close contact with a friend who was diagnosed later for about 10 minutes on July 20.
168	M, 46	28 July	Tradesman	Qilimen Community, Qilin Subdistrict, Jiangning District	He lived mainly in the store from July 13 to 22. At 12:50 on July 20, he had close contact with a customer who was diagnosed later.
169	M, 62	28 July	Retired	Chengguangxinyuan Community, Qinhui District	Between July 11 and 25, his main area of activity was near his current address.
170	M, 69	28 July	Retired	Waicaofang Community, Lukou Subdistrict, Jiangning District	From July 14 to 20, he played mahjong almost every day in the Shushan chess and card room.
171	M, 29	28 July	Airline ground crew	Jiqinjiayuan Community, Jianye District	His itinerary from July 20 to 24 mainly involves his lukou Airport, his current home address, his colleagues' home (Lishui Konggangxinyuan community), etc. He was identified as a close contact on July 24.
172	M, 50	29 July	Truck driver	Xiecun Community, Lukou Subdistrict, Jiangning District	From July 14 to 20, he commuted to work in Lukou Subdistrict, Jiangning District. His wife was diagnosed on July 25
173	M, 17	29 July	Student	Yongxin apartment buildings-Chunlan,	At noon on July 20, he rode his bike to Zhangliang restaurant, No. 133 zhengyang Road, Lukou Street.

				Lukou Subdistrict, Jiangning District	
174	F, 51	29 July	Unemployed	Fengxiangxincheng Community, Yuhuatai District	From July 15 to 27, she spent most of the daytime at home, and often went to a mahjong stall near Hongqi square in the evening. At 11:00 on July 20, she drove to Lukou Yongxing community to meet friends.
175	F, 50	29 July	Community cleaner	Daqiao Subdistrict, Gulou District	On July 26, her son was diagnosed.
176	F, 51	29 July	Unemployed	Chenggongcun Community, Lukou Subdistrict, Jiangning District	From July 15 to 20, she worked part-time at Fuyun restaurant (No. 122 Maoting Road, Lukou Street).
177	M, 25	29 July	Turck driver	Chenggongcun Community, Lukou Subdistrict, Jiangning District	Due to his work, the patient did not have a fixed track of activities, and spent most of his time delivering goods in stores and supermarkets in Lukou and surrounding areas.
178	F, 66	29 July	Peasant	Qianjia Community, Hushu Subdistrict, Jiangning District	He communicated with a neighbour who was confirmed as a case later at around 20:00 on July 24.
179	F, 65	29 July	Peasant	Baiyunlu Community, Lukou Subdistrict, Jiangning District	After July 15, her activities concentrated in the Baiyunlu community near home.
180	F, 78	29 July	Peasant	Baiyunlu Community, Lukou Subdistrict, Jiangning District	From July 14 to 20, she went out for a walk every morning, walked to Baiyun Road Senior Citizen Activity Center at noon, and often walked to Baiyun Road Haoyouduo supermarket at night.
181	F, 17	29 July	Staff	Staff of a industry factory	From July 13 to July 27, she spent most of her time at her residence and factory. On July 18, she went shopping in Yiwu commodity City by a relative's car.
182	F, 41	29 July	Cleaner of Lukou airport	Xiaopengcun Community, Lukou Subdistrict, Jiangning District	From July 14 to July 20, she spent most of her time returning to work at the airport.
183	F, 2	29 July	Infant	Waicaofang Community, Lukou Subdistrict, Jiangning District	She is the daughter of a diagnosed patient.
184	M, 27	29 July	Courier service worker	Yongxin apartment buildings-Haitang, Lukou Subdistrict, Jiangning District	From July 10 to 19, he worked mainly at the Courier station

185	F, 66	30 July	Tradesman	Waicaofang Community, Lukou Subdistrict, Jiangning District	From July 15 to 20, she went to the vegetable market at the north gate to buy vegetables every morning, and the rest of the time she stayed with her son without contact with others
186	F, 56	30 July	Owner of chess and cards room	Zhougang Community, Hushu Subdistrict, Jiangning District	She spent most of July 15-21 in the card room. At 10:40 on July 24, she went to Zhougang community vegetable market to buy vegetables.
187	M, 41	30 July	Plumber	Xiecun Community, Lukou Subdistrict, Jiangning District	He was identified as a close contact on July 22.
188	F, 30	30 July	Unemployed	Shinian Community, Lukou Subdistrict, Jiangning District	At 18:00 on July 20, she took her son to Chenhong swimming gym.
189	F, 32	30 July	E-commerce practitioner	Wenxinyuan, Molin Subdistrict, Jiangning District	She began home quarantine on July 21 after a neighbor confirmed the case.
190	F, 25	30 July	Flight attendant	Qunli Community, Lukou Subdistrict, Jiangning District	From July 16 to 20, she commuted to work at the airport. On July 21 and 23, she went shopping at Baili supermarket.
191	M, 65	31 July	Retired	Fengxiangxincheng, Tiexinqiao Subdistrict, Yuhuatai District	At 11:00 on July 18, he went to Laoshan Forest Park with his family, at 16:00 he arrived at Jiangbei Hongyang Square, and at 19:00 he arrived at Wal-mart on Daqiao North Road. He went to metro Supermarket at 8:00 on July 22. At 10:30 on July 25, he went shopping in Fengxiangxincheng Suguo supermarket.
192	F, 38	31 July	Tradesman	Liwaicheng Community, Lukou Subdistrict, Jiangning District	At 10:00 on July 17th, she went to Baihua Photography studio. At 13:00, she went to Baili Supermarket to watch movies and have dinner. At 18:00, she went to Chenghong swimming gym to swim. At 7:00 on July 21, she went to Maoting Road Tangbao Restaurant for breakfast, and then went to Baiyun Road Haoyouduo Supermarket.
193	F, 55	31 July	Umemployed	Zangjiacun Community, Lukou Subdistrict, Jiangning District	At 6:00 on July 20, she went to Lukou Shengzhuang Vegetable Market to buy vegetables and did not go out at other times. From 6:00 to 7:00 on July 21, she went shopping at Lukou Market (Lantian Road).
194	M, 14	31 July	Student	Xiaopengcun Community, Lukou Subdistrict, Jiangning District	As his parents work at Lukkou Airport, he contact with confirmed cases.
195	M, 7	31 July	Student	Zangjiacun Community, Lukou Subdistrict, Jiangning District	He was at home from 17 to 18 July. From 18:00 to 19:30 on July 19, he attended class at No.1 Gymnasium (No. 1 Huashang Road).
196	F, 42	31 July	Cleaner of Lukou airport	Kunpeng road, Jiangning District	She went to work at Lukou Airport from July 17 to 19. On July 20, she went shopping in Haoyouduo supermarket on Baiyun road.

197	M, 2	31 July	Infant	Baiyunlu Community, Lukou Subdistrict, Jiangning District	On July 21, he and his family lined up in Lukou Yanyang Square for nucleic acid testing, during which family members had close conversations with neighbors, and contacted with confirmed cases. On the evening of 23 July, he contacted with another neighbour who was a confirmed case in the backyard of his home with his family.
198	F, 55	31 July	Unemployed	Baiyunlu Community, Lukou Subdistrict, Jiangning District	She was quarantined at home from 26 to 31 July, with no visitors except nucleic acid collection staff.
199	M, 53	31 July	Staff of a company	Baiyunlu Community, Lukou Subdistrict, Jiangning District	He was quarantined at home from 26 to 31 July, with no visitors except nucleic acid collection staff.
200	M, 52	31 July	Staff of a company	Chenggongcun Community, Lukou Subdistrict, Jiangning District	From July 16 to July 20, he took the company shuttle bus to work in Shinian Community every day.
201	M, 4	31 July	Infant	Chenggongcun Community, Lukou Subdistrict, Jiangning District	He is the son of a confirmed case.
202	M, 54	31 July	Staff of Lukou airport	Zijinmingzhu, Qinhuai District	From July 18 to 22, he worked at the airport most of the time. On the night of July 17, he drove to the Banbudian Hotel for dinner.
203	F, 3	31 July	Infant	Yongxin Community, Lukou Subdistrict, Jiangning District	She is the granddaughter of a confirmed case.
204	M, 14	31 July	Student	No.10 Daqiaonan road, Yijianngmen Subdistrict, Gulou District	He is the son of the confirmed case and has the same travel schedule as the confirmed case.
205	M, 43	1 August	Teacher	Keyuan Community, Chunhua Subdistrict, Jiangning District	At 16:50 on July 19, he and his family took a taxi to Jinyuanbao Hotel for dinner. At 17:57 on July 20, he took a taxi to dinner in Yeshanghai Hotel.
206	M, 56	1 August	Staff of a company	Shinian Community, Lukou Subdistrict, Jiangning District	On July 18, he went to the Shinian brine food shop at Tongshan community to do part-time work.
207	M, 7	1 August	Infant	Shinian Community, Lukou Subdistrict, Jiangning District	He is the son of a confirmed case. He went to Chenhong swimming gym at 17:30 on July 20.
208	M, 14	1 August	Student	Zhonglvyuan, Lishui District	On the evening of July 20, 21 and 23, he went to his friend's home for dinner where he contacted with confirmed cases.

209	F, 16	1 August	Student	Xiecun Community, Lukou Subdistrict, Jiangning District	Her relatives among whom had a confirmed case came to her house for dinner on 18 July. July 19 and 20, she stayed at home.
210	F, 3	1 August	Infant	Cuipingcheng Community, Lukou Subdistrict, Jiangning District	She is the daughter of a confirmed case and has no separate itinerary.
211	M, 51	1 August	Truck driver	Waicaofang Community, Lukou Subdistrict, Jiangning District	At 13:50 on July 20, he went tBaili supermarket.
212	M, 78	1 August	Peasant	Waicaofang Community, Lukou Subdistrict, Jiangning District	From 18 July to 25 July he stayed at home with no visitors, sometimes his son who is a confirmed case came to the house.
213	M, 77	1 August	Retired	Yongxin apartmen buildings-Haitang, Lukou Subdistrict, Jiangning District	After he retired, he spent more time at home and went out less. On the morning of July 19, he went to his daughter's house in Lukou Leather City. He was identified as a close contact on July 28.
214	M, 20	1 August	Unemployed	Maoting Community, Lukou Subdistrict, Jiangning District	On July 20, he was quarantined at home after coming into contact with people who had tested positive for nucleic acid.
215	M, 41	1 August	Courier service worker	Chenggong Community, Lukou Subdistrict, Jiangning District	He worked in the logistics park every day from July 18 to July 20.
216	M, 34	2 August	Staff of Lukou airport	Ruijing Subdistrict, Qinhuai District	From July 19 to 22, he worked at the airport most of the time.
217	M, 75	2 August	Peasant	Ningguang Community, Hengxi Subdistrict, Jiangning District	He remained at home from 19 to 25 July except for nucleic acid tests.
218	F, 55	2 August	Unemployed	Yongxin Community, Lukou Subdistrict, Jiangning District	She mainly stayed at home from July 19 to 21, and went shopping at Shijihualian supermarket at19:00 on July 20. She was indentified as a close contact on July 22.
219	M, 66	2 August	Retired	Qunli Community, Lukou Subdistrict, Jiangning District	At 15:00 on July 24, he went shopping at Shijihualian supermarket. He was identified as a close contact on July 28.
220	F, 49	2 August	Peasant	Baiyunlu Community, Lukou Subdistrict, Jiangning District	On the evening of July 19, she went to Lukou Community Health Service Center. On July 20, she picked up a friend at the gate of Maoting Road Kindergarten and went to Ruyi Lake Scenic Spot for a walk.

221	F, 6	3 August	Infant	Liwaicheng Community, Lukou Subdistrict, Jiangning District	She is the daughter of the confirmed case and has the same travel schedule as the confirmed case.
222	M, 38	3 August	Airline ground crew	Qunli Community, Lukou Subdistrict, Jiangning District	At 18:10 on July 19, he and his colleagues went to zhetang food stall for dinner. He worked at the airport from July 20 to July 21 at 21:30. On July 22, he went shopping at Shijihualian supermarket.
223	F, 17	3 August	Student	Yongxin Community, Lukou Subdistrict, Jiangning District	On July 19 and 20, he went to Tianan International Building to study by airport shuttle bus and subway.
224	M, 51	4 August	Plumber of Lukou airport	Maoting Community, Lukou Subdistrict, Jiangning District	He was on duty at the airport with colleagues among whom had confirmed cases on the night of July 25.
225	M, 49	4 August	Staff of a power station	Shinian Community, Lukou Subdistrict, Jiangning District	He went to his friend's house where he contacted with a confirmed case at 9:00 on July 22.
226	F, 39	4 August	Technical staff	Zhongqian Community, Dongshan Subdistrict, Jiangning District	From July 20 to 23, she mainly worked in the workplace. After work on July 221, she went to a Xinhua bookstore.
227	F, 23	4 August	Technical staff	Jiangning Community, Jiangning Subdistrict, Jiangning District	From July 20 to 21, she mainly worked in the workplace.
228	F, 72	5 August	Unemployed	Ninguang Community, Hengxi Subdistrict, Jiangning District	From July 21 to 25, she stayed at home except for nucleic acid tests.
229	F, 25	6 August	Technical staff	Xintong Community, Jiangning Subdistrict, Jiangning District	She was quarantined on 21 July.
230	F, 78	7 August	Pearant	Shinian Community, Lukou Subdistrict, Jiangning District	From 23 July to 3 August, she was quarantined at home and had no contact with outsiders except for nucleic acid tests. She was identified as a close contact on August 4.
231	F, 50	7 August	Unemployed	Shinian Community, Lukou Subdistrict, Jiangning District	From 23 July to 3 August, she was quarantined at home and had no contact with outsiders except for nucleic acid tests. She was identified as a close contact on August 4.
232	F, 4 months	9 August	Infant	Shinian Community, Lukou Subdistrict, Jiangning District	She goes with her family every day without single track. She was identified as a close contact on August 4.

233	M, 81	9 August	Unemployed	Shinian Community, Lukou Subdistrict, Jiangning District	From 25 July to 3 August, he was quarantined at home and had no contact with outsiders except for nucleic acid tests. He was identified as a close contact on August 4.
234	M, 32	11 August	Doctor	–	On 29 July, he was admitted to the Nanjing Public Health And Medical Center and used a special vehicle to commute from his workplace to his designated residence
235	F, 26	12 August	Kindergarener	Shinian Community, Lukou Subdistrict, Jiangning District	From 28 July to 4 August, she was quarantined at home. She was identified as a close contact on August 4.

2 Supplementary Figures and Tables

2.1 **Supplementary Figure 1.** The network used for analysis in the study.

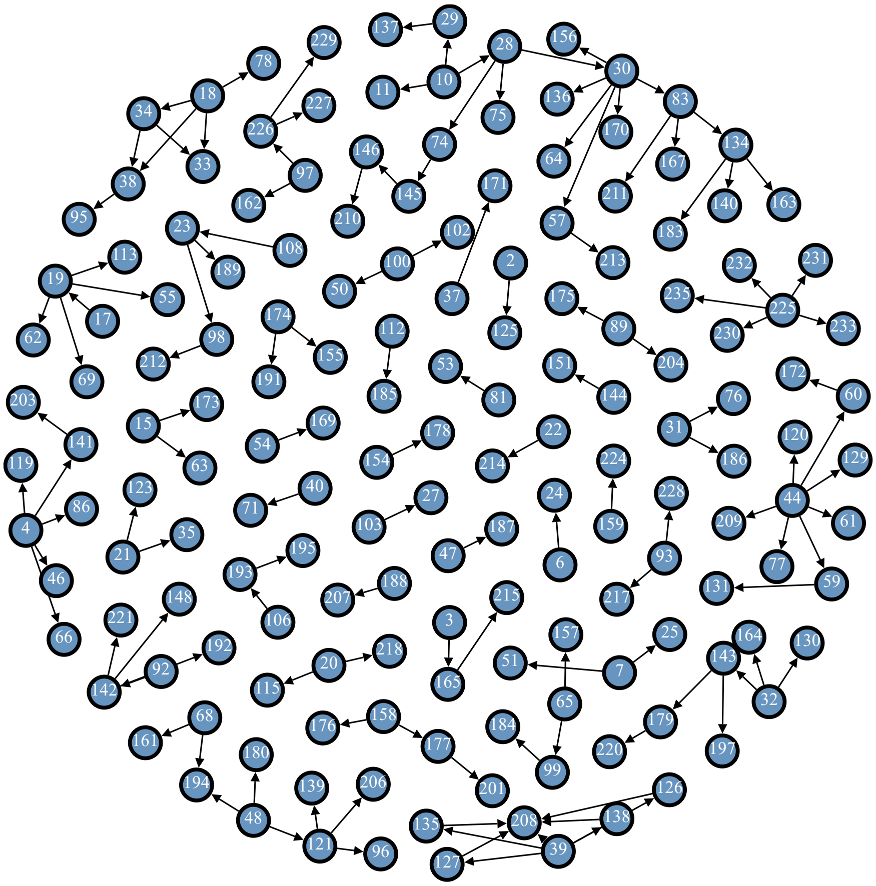

2.2 **Supplementary Figure 2.** Statistics of Components initiation date. Component initiation date is the diagnosis date of the primary patient in that component.

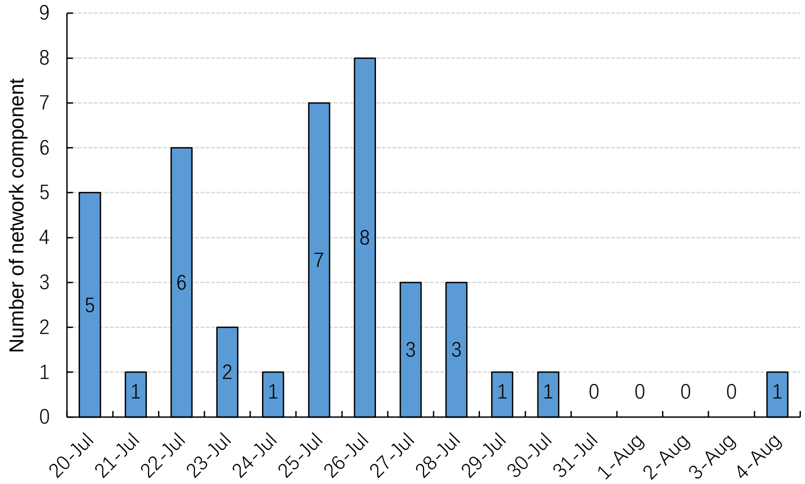

Supplement: Supplementary file 1 [file Data_Sheet_1.PDF]
